# Time trends in mortality of congenital heart disease in children aged 0–14 years: a global, regional, and national cohort analysis from 1990 to 2021 using the global burden of disease study

**DOI:** 10.3389/fpubh.2025.1537671

**Published:** 2025-07-02

**Authors:** Jiaoli Xu, Qinhong Li, Jingxuan Xiong, Zugen Cheng, Lili Deng

**Affiliations:** ^1^Department of Cardiology, Kunming Children's Hospital, Kunming, China; ^2^Department of Cardiology, The First Affiliated Hospital of Kunming Medical University, Kunming, Yunnan, China; ^3^Department of Respiratory Medicine, Kunming Children's Hospital, Kunming, Yunnan, China

**Keywords:** mortality, childhood, congenital heart disease (CHD), public health, GBD (global burden disease)

## Abstract

**Introduction:**

Congenital heart disease (CHD) represents a significant global public health burden, with substantial variability in mortality rates across different regions and age groups.

**Methods:**

This study utilized the Global Burden of Disease (GBD) database to examine trends in CHD-related mortality among children aged 0-14 from 1990 to 2021.

**Results:**

We report a 55.34% reduction in CHD-related deaths among children, with global mortality rates decreasing from 28.63 per 100,000 in 1990 to 11.06 per 100,000 in 2021. Notably, the decline in mortality was more pronounced in younger children, with the highest burden observed in the Low socio-demographic index (SDI) region, where CHD-related mortality rates remain disproportionately high. In contrast, the high SDI region experienced the greatest improvements in mortality reduction. Regional disparities are also evident, with South Asia bearing the highest number of CHD-related deaths, while Oceania exhibited the highest mortality rate.

**Discussion:**

These trends underscore the need for continued global efforts to reduce CHD-related mortality, particularly in low-income regions, and to address the disparities in healthcare access and outcomes. Our findings highlight the ongoing challenges in pediatric cardiology and the need for targeted interventions to sustain improvements in CHD survival, especially for neonates and infants.

## Introduction

Congenital heart disease (CHD), also referred to as congenital heart defects, encompasses a group of congenital anomalies of the cardiovascular system arising from abnormal or disrupted cardiac and large vessel development during early embryogenesis (1). This diverse array of conditions, ranging from simple to complex malformations, represents the most common birth defect, affecting ~1% of live births (2) and contributing to 30% of fetal deaths (3). Despite extensive research to elucidate the origins of CHD, the causes of most cases remain elusive. However, advances in cardiac diagnostics, and interventional, and surgical techniques have led to notable declines in CHD mortality (4).

Globally, a significant decline in CHD-related mortality has been observed. For example, a Canadian cohort study reported a 30% decrease in all-cause mortality among CHD patients between 1987 and 2005 (5). Similarly, Lopez and colleagues demonstrated declining trends in overall CHD-attributed mortality in the United States (a 39% reduction), while highlighting persistent racial and ethnic disparities (6). These disparities are often compounded by socioeconomic inequalities in access to prenatal screening, specialized care, and post-operative rehabilitation services. Globally, the crude mortality rate for CHD fell from 7.1 per 100,000 in 1990 to 2.8 per 100,000 in 2019 (7). Data from Finland also indicated reduced early and late postoperative CHD mortality from 1990–2009 compared to the 1953–1989 period (8). Data from Belgium and Sweden suggest that a significant proportion of children born with CHD since the 1970s have survived into adulthood (9, 10). However, such progress remains unevenly distributed, with lower- and middle-income countries facing disproportionately high mortality rates due to limited healthcare infrastructure and financial barriers.

Although CHD-related mortality has declined overall, CHD remains a highly heterogeneous condition. It includes both simple defects that require minimal intervention and more complex anomalies that demand intensive medical and surgical treatment from birth. Globally, an estimated 1.35 million newborns are affected by CHD each year (11), while in the United States, ~2.4 million individuals are currently living with CHD (12). Annually, CHD accounts for around 250,000 deaths globally (13). A national database study focused on neonates with hypoplastic left heart syndrome (HLHS) observed a 20% decline in mortality from 1998–2005 to 2006–2014 (14). Yet, over the past decade, declines in CHD mortality rates have started to plateau. Despite ongoing advancements in surgical techniques and critical care, neonatal mortality associated with severe CHD remains alarmingly high. Recent estimates indicate that ~24.5% of neonatal deaths are attributable to CHD (15).

These trends highlight the significant burden of CHD on affected individuals and their families, particularly in resource-limited settings where socioeconomic inequalities amplify disease severity and mortality risk. The Global Burden of Disease (GBD) Study reported 217,000 CHD-related deaths worldwide in 2019 (16), emphasizing the importance of reducing CHD mortality to mitigate the broader burden of noncommunicable diseases. There have been no comprehensive studies of long-term global epidemiological trends in childhood CHD. Here, we utilize the GBD database to analyze trends in CHD-related mortality among children aged 0–14 from 1990 to 2021. It is anticipated that insights from GBD 2021 estimates will contribute to a better understanding of the CHD burden, thereby guiding public health initiatives to alleviate this condition.

## Methods

### Overview and data collection

This study was approved by the Ethical Committee of Kunming Children's Hospital, which granted a waiver of informed consent since it involved only analysis of de-identified data. Data on CHD among children aged 0–14 years—including standardized disease definitions and mortality information—were obtained from the Global Health Data Exchange Query Tool developed by the GBD collaborators (https://vizhub.healthdata.org/gbd-results/). We collected data on CHD-related mortality cases and corresponding rates at global, regional, and national levels. As the GBD database does not collect information on participants' race and ethnicity, such data were not included in our analysis. The average estimated annual percentage change (EAPC) was calculated using linear regression (17). Our study adhered to the Strengthening the Reporting of Observational Studies in Epidemiology (STROBE) guidelines (18).

### Sociodemographic index

This study aims to evaluate the socio-economic development levels of countries and regions through the sociodemographic index (SDI), a composite measure based on total fertility rate, average years of education, and per capita income (19). The SDI ranges from 0 to 1, with higher values indicating greater socio-economic development. Based on the SDI, countries and regions were classified into five levels: low, low-middle, middle, middle-high, and high, to explore the relationship between socio-economic development and the mortality burden of CHD in children. CHD mortality data were obtained from the GBD database, and countries and regions were categorized according to their SDI values during the study period. We further conducted a comparative analysis of CHD mortality rates across different SDI levels to reveal the impact of socio-economic development disparities on CHD mortality in children.

### Statistical analysis

Mortality rates and their corresponding ratios serve as key indicators of the burden of CHD in children. According to the GBD methodology, incidence rates are reported per 100,000 individuals, along with 95% uncertainty intervals (UIs). The EAPC was calculated to evaluate temporal trends in the burden of pediatric CHD. Linear modeling was subsequently employed to determine the 95% confidence interval (CI) of the EAPC (20). If both the upper and lower bounds of the 95% CI for the EAPC are negative, it indicates a decreasing trend in mortality. Conversely, if both bounds are positive, it suggests an increasing trend in mortality (21).

## Results

### Leading causes of mortality in children under 14 years of age attributed to congenital anomalies, 1990–2021

**Figure 1:**
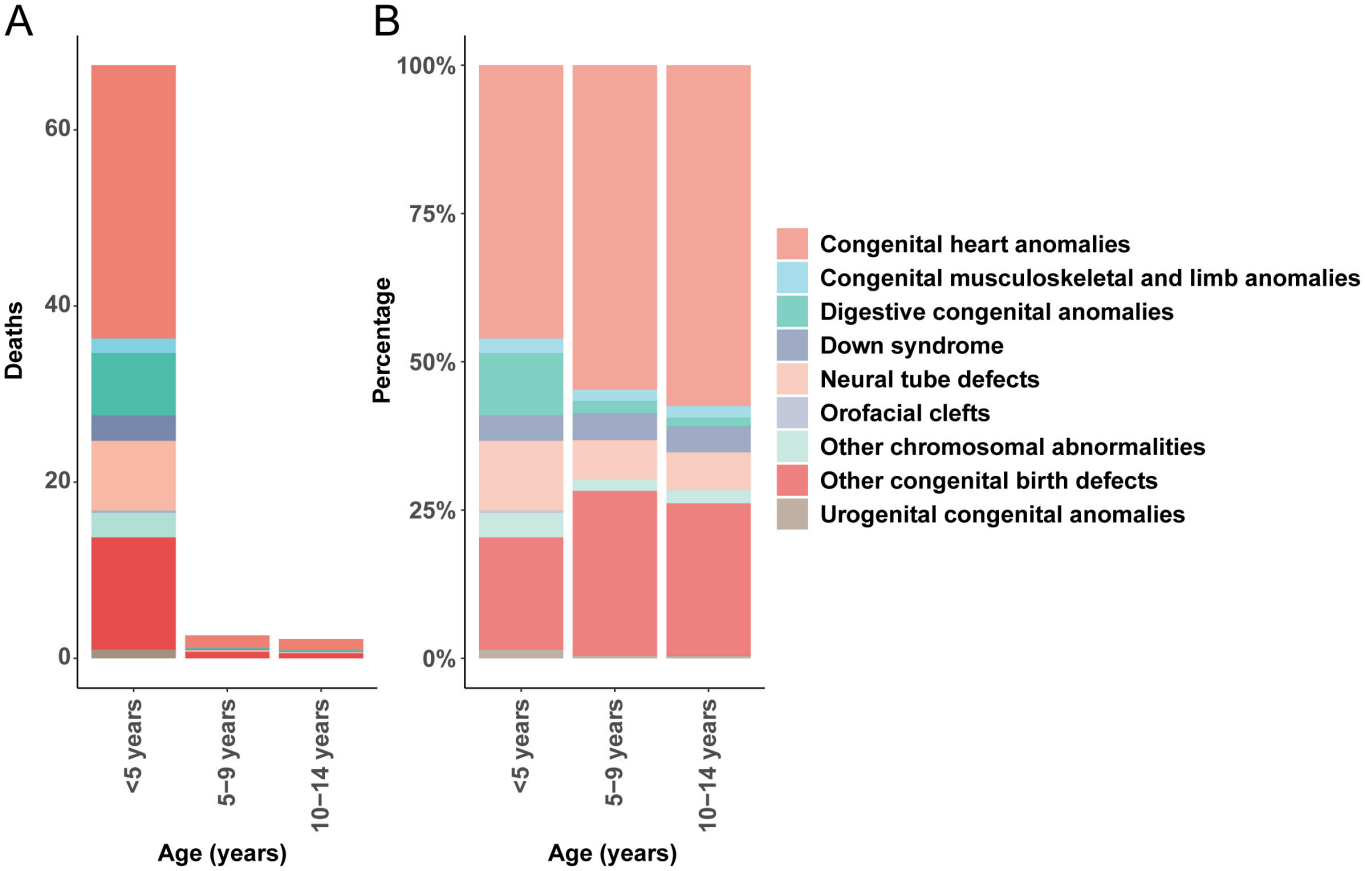
Age-specific mortality rates and proportional contributions of congenital anomalies. **(A)** Death rate. **(B)** Death percentage.

In 2021, congenital anomalies posed a significant burden on global child mortality. Among children under the age of five, CHD accounted for the highest mortality rate (31.03; 95% UI, 25.11–38.81), whereas orofacial clefts had the lowest (0.26; 95% UI, 0.07–0.67). In the 5–9-year age group, CHD remained the leading cause of death due to congenital anomalies (1.43; 95% UI, 1.19–1.77), while urogenital congenital anomalies had the lowest mortality (0.01; 95% UI, 0.01–0.02). A similar trend was observed among 10–14-year-olds, with CHD again exhibiting the highest mortality rate (1.26; 95% UI, 1.07–1.55), while urogenital congenital anomalies had the lowest rate (0.01; 95% UI, 0.01–0.01; Figure 1).

These findings emphasize that CHD consistently represents the highest mortality rate among congenital birth defects in children under 14 years. Importantly, the mortality rate associated with CHD demonstrated a declining trend with increasing age, underscoring the heightened vulnerability of younger children to these conditions.

### Global trends in CHD mortality, 1990–2021

In 2021, the global number of CHD-related deaths among children under 14 years of age was 222,415.18 (95% UI, 181,358.96–275,182.31), indicating a substantial reduction of 55.34% (23.46–65.60) from 497,979.20 (95% UI, 282,165.57–642,052.05) in 1990. Notably, the mortality rate due to CHD decreased from 28.63 (95% UI, 16.22–36.92) per 100,000 in 1990 to 11.06 (95% UI, 9.01–13.68) per 100,000 in 2021, with an EAPC of −2.57 (95% CI, −2.74 to −2.41; Table 1). This trend highlights substantial progress in pediatric healthcare and interventions over the past three decades.

**Table 1:** Global and regional burden of congenital heart disease mortality in children under 14 years between 1990 and 2021.

**Location**	**1990 (95% UI)**		**2021 (95% UI)**		**Cases change**	**EAPC^a^**
	**Deaths cases**	**Deaths rate**	**Deaths cases**	**Deaths rate**		
Global	497,979.20 (282,165.57, 642,052.05)	28.63 (16.22, 36.92)	222,415.18 (181,358.96, 275,182.31)	11.06 (9.01, 13.68)	−55.34 (−65.60, −23.46)	−2.57 (−2.74, −2.41)
High SDI	19,278.02 (16,530.66, 21,072.99)	10.38 (8.90, 11.34)	4,082.73 (3,323.73, 4,958.15)	2.37 (1.93, 2.87)	−78.82 (−83.26, −72.45)	−4.30 (−4.50, −4.10)
High-middle SDI	74,119.96 (51,730.46, 91,930.31)	27.09 (18.91, 33.60)	11,657.25 (9,504.67, 14,097.48)	5.05 (4.12, 6.11)	−84.27 (−88.41, −74.88)	−4.87 (−5.15, −4.59)
Middle SDI	161,973.93 (99,771.51, 214,783.75)	28.06 (17.29, 37.21)	45,808.92 (37,937.52, 56,010.44)	8.08 (6.69, 9.88)	−71.72 (−79.60, −46.10)	−3.29 (−3.52, −3.06)
Low-middle SDI	152,059.30 (80,501.32, 207,295.95)	32.21 (17.05, 43.91)	74,173.96 (57,959.56, 93,683.43)	12.79 (10.00, 16.16)	−51.22 (−64.78, 5.88)	−2.50 (−2.67, −2.33)
Low SDI	90,138.59 (31,961.88, 132,359.15)	39.38 (13.96, 57.82)	86,447.02 (59,760.01, 117,370.19)	18.78 (12.98, 25.50)	−4.10 (−27.20, 94.53)	−2.18 (−2.29, −2.07)
**Regions**						
Andean Latin America	5,089.87 (2,721.57, 6,654.80)	34.27 (18.32, 44.81)	2,197.30 (1,645.21, 2,828.72)	12.14 (9.09, 15.63)	−56.83 (−70.62, −10.09)	−2.62 (−2.85, −2.39)
Australasia	247.36,(225.17, 275.87)	5.39 (4.91, 6.02)	94.57 (68.07, 119.49)	1.65 (1.19, 2.08)	−61.77 (−72.89, −51.55)	−3.34 (−3.60, −3.09)
Caribbean	3,819.15 (2,887.57, 4,817.15)	33.46 (25.30, 42.21)	2,386.60 (1,480.83, 3,944.34)	20.74 (12.87, 34.28)	−37.51 (−57.37, 4.17)	−1.11 (−1.40, −0.83)
Central Asia	4,022.44 (3,461.62, 4,563.80)	16.10 (13.85, 18.26)	4,284.33 (3,344.08, 5,336.06)	15.48 (12.08, 19.28)	6.51 (−14.42, 31.63)	0.92 (0.61, 1.23)
Central Europe	4,746.81 (4,050.35, 5,291.02)	16.10 (13.74, 17.95)	666.39 (537.45, 788.14)	3.76 (3.04, 4.45)	−85.96 (−89.64, −82.71)	−4.28 (−4.41, −4.15)
Central Latin America	11,547.42 (10,113.01, 13,189.37)	17.94 (15.71, 20.49)	7,498.30 (5,749.45, 9,612.47)	11.81 (9.06, 15.14)	−35.07 (−51.53, −13.73)	−0.97 (−1.22, −0.73)
Central Sub-Saharan Africa	7,625.78 (2,259.92, 13,924.86)	30.14 (8.93, 55.04)	6,126.04 (3,809.62, 9,930.28)	10.44 (6.49, 16.92)	−19.67 (−42.83, 92.55)	−2.98 (−3.31, −2.64)
East Asia	116,856.43 (72,956.38, 162,097.09)	35.43 (22.12, 49.15)	14,478.92 (11,062.88, 18,949.03)	5.42 (4.14, 7.09)	−87.61 (−91.90, −76.35)	−5.32 (−5.62, −5.02)
Eastern Europe	7,149.89 (6,294.60, 8,669.64)	13.89 (12.23, 16.85)	1,200.87 (965.51, 1,509.94)	3.39 (2.72, 4.26)	−83.20 (−87.82, −77.70)	−3.53 (−4.42, −2.64)
Eastern Sub-Saharan Africa	28,236.43 (7,624.44, 54,649.63)	31.18 (8.42, 60.34)	22,426.76 (13,651.88, 40,236.17)	12.57 (7.65, 22.55)	−20.58 (−45.11, 106.34)	−2.67 (−2.79, −2.56)
High-income Asia Pacific	3,361.71 (2,689.73, 3,821.10)	9.55 (7.64, 10.86)	367.58 (281.22, 495.19)	1.64 (1.25, 2.21)	−89.07 (−91.16, −82.43)	−5.35 (−5.54, −5.17)
High-income North America	4,910.01 (4,186.40, 5,383.74)	7.96 (6.79, 8.73)	1,598.78 (1,323.10, 1,996.92)	2.44 (2.02, 3.04)	−67.44 (−73.83, −56.01)	−3.24 (−3.48, −3.00)
North Africa and Middle East	94,197.46 (41,643.56, 133,933.60)	67.05 (29.64, 95.34)	33,239.69 (26,354.03, 41,207.74)	18.13 (14.38, 22.48)	−64.71 (−74.71, −31.11)	−3.56 (−3.81, −3.30)
Oceania	981.87 (336.63, 1,465.71)	36.64 (12.56, 54.69)	1,651.69 (679.22, 2,509.70)	32.51 (13.37, 49.40)	68.22 (27.94, 135.62)	−0.22 (−0.36, −0.07)
South Asia	110,569.39 (69,282.14, 148,838.95)	25.51 (15.99, 34.35)	51,311.96 (36,235.38, 72,898.22)	10.12 (7.15, 14.38)	−53.59 (−68.41, 1.50)	−2.53 (−2.69, −2.38)
Southeast Asia	43,886.43 (20,947.85, 59,470.11)	25.70 (12.27, 34.83)	20,342.99 (16,612.67, 25,296.64)	11.78 (9.62, 14.65)	−53.65 (−66.15, −1.74)	−2.41 (−2.53, −2.29)
Southern Latin America	2,027.10 (1,687.38, 2,398.01)	13.58 (11.30, 16.07)	860.56 (694.66, 1,058.04)	5.94 (4.79, 7.30)	−57.55 (−67.77, −44.90)	−2.00 (−2.36, −1.64)
Southern Sub-Saharan Africa	1,802.11 (1,461.69, 2,353.87)	8.71 (7.06, 11.38)	1,486.40 (976.98, 2,014.74)	6.18 (4.06, 8.37)	−17.52 (−42.11, 19.74)	−0.52 (−0.71, −0.32)
Tropical Latin America	8,050.03 (6,812.99, 9,340.62)	15.01 (12.71, 17.42)	4,653.01 (3,700.77, 5,710.17)	9.27 (7.37, 11.38)	−42.20 (−56.69, −24.64)	−0.77 (−1.16, −0.37)
Western Europe	6,132.21 (5,246.36, 6,694.04)	8.63 (7.39, 9.43)	1,357.54 (1,068.19, 1,674.87)	1.99 (1.57, 2.46)	−77.86 (−83.21, −70.92)	−4.55 (−4.71, −4.38)
Western Sub-Saharan Africa	32,719.31 (82,84.07, 50,360.46)	37.23 (9.43, 57.31)	44,184.91 (25,693.22, 62,761.45)	20.57 (11.96, 29.22)	35.04 (2.51, 231.80)	−1.52 (−1.69, −1.36)

EAPC, estimated annual percentage change; SDI, sociodemographic index; UI, uncertainty interval. EAPC^a^ is expressed as 95% CIs.

In 2021, CHD-related deaths among children under 5 years of age were reported at 204,222.9 (95% UI, 165,238.47–255,409.19), representing a 56.19% (24.74–66.44) decline from 466,156.65 (95% UI, 261,282.55–601,016.42) in 1990. The mortality rate decreased from 75.19 (95% UI, 42.15–96.95) per 100,000 in 1990 to 31.03 (95% UI, 25.11–38.81) per 100,000 in 2021, with an EAPC of −2.58 (95% CI, −2.70 to −2.47; Supplementary Table S1). This significant decline may be attributed to advancements in early diagnosis, improved surgical techniques, and enhanced access to pediatric cardiac care.

For children aged 5–9 years, CHD-related deaths in 2021 were reported at 9,824.63 (95% UI, 8,141.66–12,141.51), indicating a 51.13% (16.43–62.42) reduction from 20,102.32 (95% UI, 12,638.47–25,808.87) in 1990. Correspondingly, the mortality rate dropped from 3.44 (95% UI, 2.17–4.42) per 100,000 in 1990 to 1.43 (95% UI, 1.19–1.77) per 100,000 in 2021, with an EAPC of −2.61 (95% CI, −2.75 to −2.46; Supplementary Table S2). This trend underscores the effectiveness of ongoing efforts to improve pediatric healthcare outcomes for school-aged children.

Among children aged 10–14 years, there were 8,367.58 (95% UI, 7,122.57–10,337.39) CHD-related deaths in 2021, representing a 28.61% (3.65–42.54) reduction from 11,720.23 (95% UI, 8,561.76–14,300.85) in 1990. The mortality rate decreased from 2.19 (95% UI, 1.60–2.67) per 100,000 in 1990 to 1.26 (95% UI, 1.07–1.55) per 100,000 in 2021, with an EAPC of −1.64 (95% CI, −1.73 to −1.56; Supplementary Table S3).

### Congenital heart disease in children: SDI regional trends

**Figure 2:**
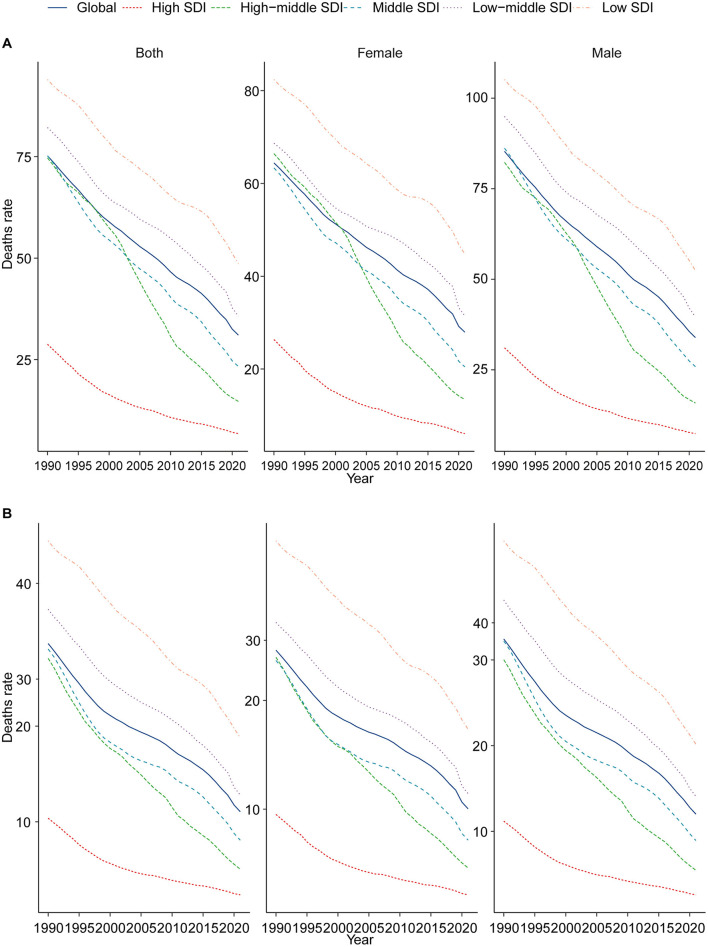
Epidemiologic trends in childhood CHD mortality across five SDI regions (1990–2021). **(A)** Trends in children aged 0–5; **(B)** trends in children aged 0–14.

In 1990, the highest number of CHD-related deaths among children aged 0–14 years was recorded in the middle SDI region, with an estimated 161,973.93 deaths (95% UI, 99,771.51–214,783.75). In contrast, the high SDI region recorded the lowest number of CHD-related deaths, at 19,278.02 (95% UI, 16,530.66–21,072.99). Notably, by 2021, the region with the highest number of CHD-related deaths among children aged 0–14 years had shifted to the low SDI region, with 86,447.02 deaths (95% UI, 59,760.01–117,370.19). The high SDI region continued to report the lowest number of deaths, at 4,082.73 (95% UI, 3,323.73–4,958.15). The CHD mortality rate was highest in the low SDI region, at 18.78 per 100,000 (95% UI, 12.98–25.50), while the high SDI region had the lowest rate, at 2.37 per 100,000 (95% UI, 1.93–2.87). Of particular interest, the high-middle SDI region experienced the lowest annual percentage change in CHD-related mortality (EAPC), at −4.87 (95% CI, −5.15 to −4.59). In 2021, both boys and girls aged 0–14 years exhibited the highest CHD mortality rates in the low SDI region (Table 1 and Figure 2B).

Among children aged 0–5 years, CHD-related mortality declined significantly between 1990 and 2021 across three of the five SDI regions: high SDI, high-middle SDI, and middle SDI, each showing reductions exceeding 70%. However, the low SDI region experienced only a modest decrease of 5.58%. The high-middle SDI region recorded the lowest EAPC, at −5.31 (95% CI, −5.65 to −4.97). In 2021, CHD mortality rates among both boys and girls aged 0–5 years were highest in the low SDI region (Supplementary Table S1 and Figure 2A). For children aged 5–9 years in 2021, the highest number of CHD-related deaths was observed in the low SDI region, with 3,311.10 deaths (95% UI, 2,504.89–4,452.94), whereas the high SDI region had the lowest number of deaths, at 214.33 (95% UI, 173.33–262.14). The high-middle SDI region also exhibited the lowest EAPC, at −4.15 (95% CI, −4.30 to −3.99). In 2021, the CHD mortality rates for boys and girls aged 5–9 years were highest in the low SDI region (Supplementary Table S2 and Supplementary Figure S1A). Finally, in children aged 10–14 years in 2021, CHD-related deaths were most prevalent in the low-middle SDI region, with 2,735.81 deaths (95% UI, 2,242.86–3,535.26). By comparison, the high SDI region had the lowest number of deaths, at 230.00 (95% UI, 202.56–279.28), and also recorded the lowest EAPC, at −3.52 (95% CI, −3.67 to −3.37). Importantly, the CHD mortality rates for boys and girls aged 10–14 years remained highest in the low SDI region (Supplementary Table S3 and Supplementary Figure S1B).

### CHD in children: geographic regional trends

**Figure 3:**
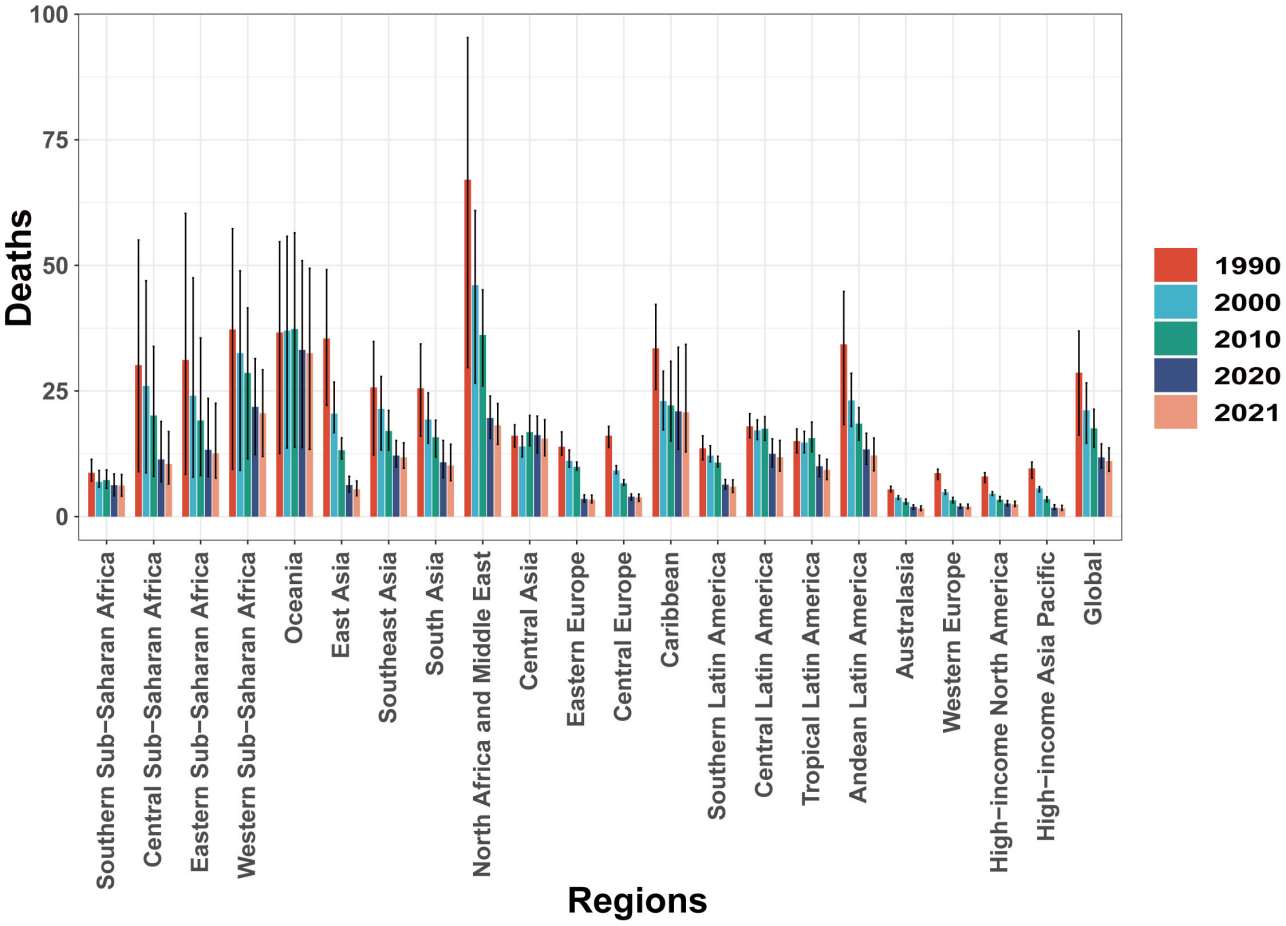
Mortality trends of childhood CHD: a global and regional analysis from 1990 to 2021.

In 2021, CHD mortality among children aged 0–14 years demonstrated substantial regional variation worldwide. Australasia reported the lowest number of CHD-related deaths, with an estimated 94.57 cases (95% UI, 68.07–119.49), while South Asia bore the highest burden, with 51,311.96 deaths (95% UI, 36,235.38–72,898.22). The lowest CHD mortality rate was observed in the High-income Asia Pacific region, at 1.64 per 100,000 population (95% UI, 1.25–2.21), whereas Oceania recorded the highest rate, at 32.51 per 100,000 population (95% UI, 13.37–49.40). The High-income Asia Pacific region notably exhibited the most significant decline in the EAPC of CHD mortality, with a value of −5.35 (95% CI, −5.54 to −5.17). In contrast, Central Asia experienced the highest EAPC, at 0.92 (95% CI, 0.61–1.23), indicating a modest increase in CHD-related deaths. In 2021, the global SDI was estimated at 0.67, with a corresponding global average CHD mortality rate of 11.06 per 100,000 population. Nine regions exhibited CHD mortality rates above the global average, whereas 12 regions had mortality rates below the global mean, underscoring disparities in the burden of CHD across different socio-demographic settings (Table 1 and Figure 2). Additionally, between 1990 and 2021, all 21 regions experienced declining mortality rates to varying extents (Figure 3).

### CHD in children: national trends

**Figure 4:**
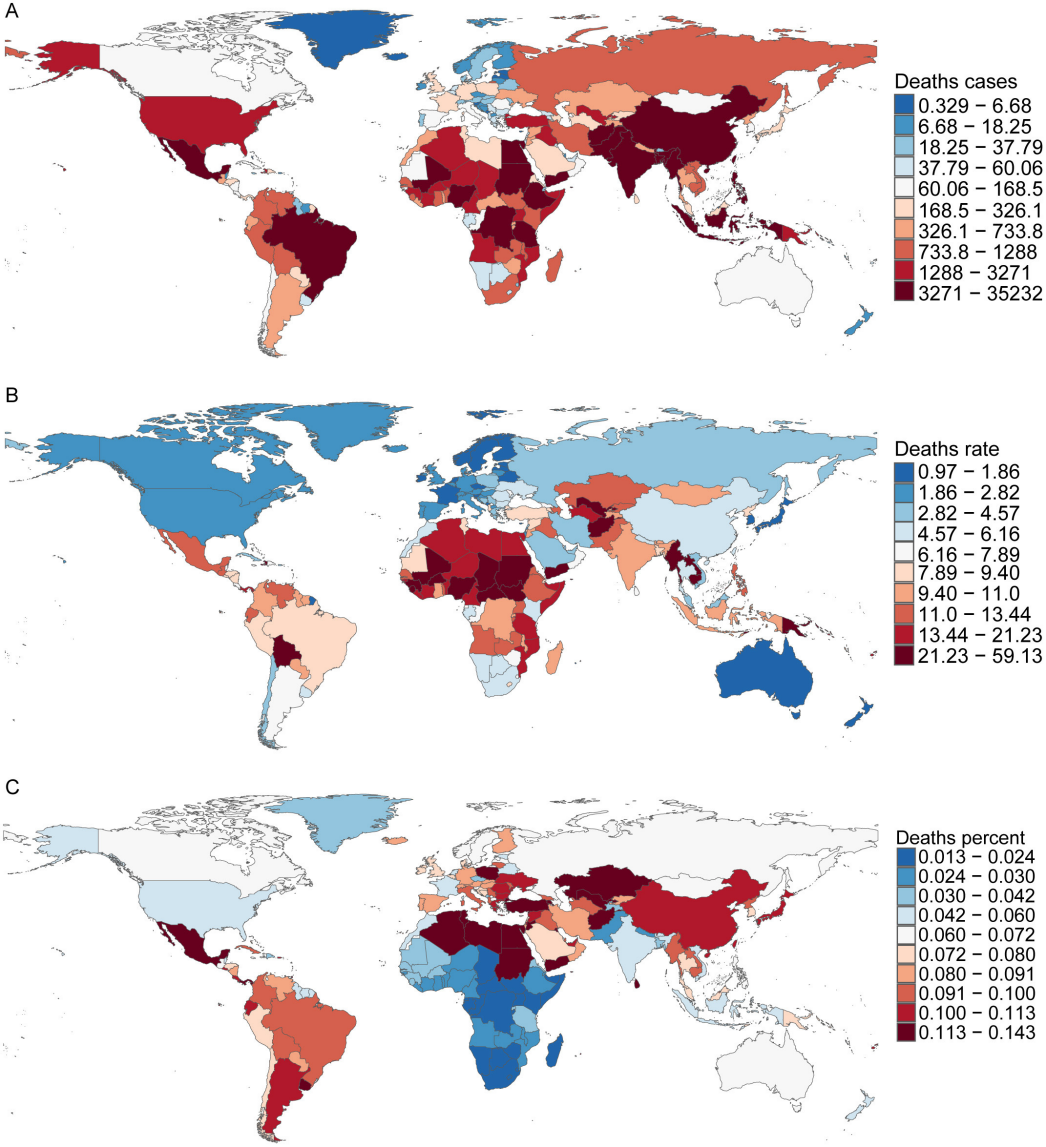
Death cases, mortality rates, and proportional burden of childhood CHD in 204 countries and territories. **(A)** Death cases; **(B)** mortality rates; **(C)** proportion.

In 2021, San Marino reported the lowest number of CHD-related deaths among children aged 0–14 years, with an estimated 0.02 cases (95% UI, 0–0.03). In contrast, India recorded the highest number of CHD-related deaths, with 35,232 cases (95% UI, 25,272–50,611). Afghanistan had the highest CHD mortality rate, 59.1 per 100,000 (95% UI, 28.98–84.46), while San Marino had the lowest mortality rate at 0.45 per 100,000 (95% UI, 0.25 to 0.80). Lesotho reported the lowest proportion of CHD deaths, accounting for just 1% of total deaths (95% UI, 0.01–0.02), whereas Panama had the highest proportion, reaching 14% (95% UI, 12%−16%). Regarding the EAPC, Saudi Arabia showed the most significant decline, with an EAPC of −7.89 (95% CI, −8.03 to −7.75), while Guatemala had the highest increase, with an EAPC of 3.23 (95% CI, 2.43–4.05; Figure 4 and Supplementary Table S4).

## Discussion

This study analyzed the mortality rates and trends of CHD among children aged 0–14 years across global regions and countries from 1990 to 2021, highlighting the current burden of CHD in areas with varying income levels. Despite a general decline in global mortality rates of pediatric CHD over the past three decades, CHD remains the leading cause of death from congenital anomalies in children under 14, particularly in low SDI regions. These findings underscore the urgency of addressing this persistent health challenge.

Our data reveal that the global number of CHD-related deaths decreased from ~498,000 in 1990 to 222,000 in 2021, with the mortality rate dropping from 28.63 to 11.06 per 100,000 population—a mean annual percentage change of −2.57%. However, this decline was not uniform across regions and countries; low-SDI areas continue to exhibit high mortality rates with limited decreases. For instance, Afghanistan recorded the highest CHD mortality rate, while San Marino had the lowest. Saudi Arabia achieved the most significant mortality reduction, whereas Guatemala experienced an increase. This disparity likely reflects differences in healthcare resource allocation and the capacity of health systems worldwide.

Advancements in medical technology have played a crucial role in reducing CHD mortality rates. High-income countries have improved cardiac diagnostic methods and developed sophisticated surgical interventions, enabling most CHD cases to receive accurate diagnosis and corrective treatment, thereby significantly enhancing prognosis (22). However, CHD exhibits substantial individual variability, especially in complex and compound malformations, necessitating personalized treatment plans based on severity and hemodynamic status. Early diagnosis and timely intervention remain paramount but are often challenging to implement in resource-limited settings (23).

Our findings align with previous studies. In 2017, 69% of all CHD-related deaths occurred in infants under 1 year old, representing a 34.5% decrease from 398,580 cases in 1990 (13). The 2019 GBD study also reported a 42.7% reduction in CHD deaths, from 378,000 in 1990 to 217,000 in 2019 (16). Additionally, heart failure remains a significant complication among CHD patients (24). Studies indicate that at least 50% of CHD patients develop right ventricular dysfunction, such as in cases of congenitally corrected transposition of the great arteries (l-TGA) (25). Wang et al. (26) reported that among 14,982 adult CHD patients aged ≥40 years in Quebec, Canada, 12.2% were hospitalized for heart failure, with 23% dying within 1 year post-admission.

Notably, some low-SDI countries have seen an upward trend in CHD mortality among children (e.g., Mali, SDI = 0.27), possibly due to limited medical infrastructure hindering early diagnosis and timely treatment. These regions may face challenges such as inadequate healthcare resources, shortage of specialized personnel, and weak health infrastructure, highlighting gaps that need urgent attention.

In this study exploring CHD's epidemiological characteristics and trends, we did not include data on pregnancy termination rates and related policies across different countries or regions, which may significantly influence CHD prevalence and outcomes. Previous studies indicate substantial variability in national and regional policies regarding pregnancy termination following prenatal diagnosis (27). Regions with restrictive pregnancy termination policies may experience higher live birth rates of severe CHD cases, thereby increasing CHD prevalence among live births. Conversely, regions with more permissive policies typically report lower prevalence and mortality rates. The absence of analysis regarding this factor may limit the generalizability and depth of our study's conclusions.

The etiology of CHD is believed to involve both genetic and environmental factors. Approximately 15% of CHD cases are attributable to single-gene mutations and chromosomal abnormalities (28). Critical transcription factors like NKX2.5, TBX5, and GATA4 play vital roles during heart development by regulating downstream gene expression; their anomalies can lead to CHD (29). Moreover, exposure to biological, chemical, or physical environmental factors during the first 8 weeks of embryonic development—when the four-chambered heart forms—can increase the risk of CHD (30). Additionally, while the precise etiology of CHD remains incompletely understood, the evidence clearly links prenatal exposure to known teratogenic factors—including alcohol, tobacco (31), certain medications (32), and environmental pollutants (33)—to increased risks of fetal CHD. For instance, maternal smoking in early pregnancy is closely associated with conditions such as tetralogy of Fallot and pulmonary valve stenosis, whereas prenatal alcohol consumption significantly elevates the risk of ventricular and atrial septal defects. Given the significance of these harmful exposures in the etiology of CHD, the current study's lack of detailed analysis regarding their specific contribution to disease burden constitutes another important limitation.

In light of these findings, reducing the global burden of CHD requires comprehensive strategies. Strengthening prenatal care and providing health education can lower CHD incidence. Simultaneously, policymakers and healthcare institutions must invest in improving pediatric cardiac care in resource-limited regions, including training specialists, enhancing medical infrastructure, and adopting appropriate medical technologies.

In conclusion, CHD remains a significant global health challenge affecting children's health, especially in low-SDI areas. Our study emphasizes the importance of continued efforts to reduce CHD-related mortality worldwide. Through collaborative, multi-sectoral interventions, it is possible further to mitigate the impact of CHD on child health.

### Limitations and future directions

While this study provides valuable insights into pediatric CHD, it has several noteworthy limitations that should be addressed in future research. Primarily, our analysis relies heavily on the GBD database, whose accuracy is constrained by factors such as the limited availability and reliability of national registry data, the substantial number of undiagnosed CHD cases in children, and the absence of information on other risk factors related to pediatric CHD. Additionally, our dataset lacks details on the specific subtypes of congenital heart disease, preventing a more granular analysis.

To overcome these limitations and enhance our understanding of the global burden of pediatric CHD, future studies should focus on improving data collection and reporting mechanisms, especially in resource-limited settings. Strengthening national registries to ensure comprehensive and accurate data capture would provide a more robust foundation for analysis. Enhancing early diagnosis and screening programs can help identify undiagnosed cases, offering a more complete picture of disease prevalence. Furthermore, obtaining detailed information on CHD subtypes and associated risk factors would contribute to a deeper understanding of the disease's etiology and progression. Addressing these challenges is essential for developing more effective public health strategies aimed at improving cardiovascular health outcomes for children worldwide.

## Conclusion

This comprehensive analysis of global trends in CHD mortality among children from 1990 to 2021 highlights significant progress in reducing CHD-related deaths, with an overall decline of 55.34%. Advances in early diagnosis, surgical interventions, and improved pediatric cardiac care have substantially decreased mortality rates across most regions.

## Data Availability

The datasets presented in this study can be found in online repositories. The names of the repository/repositories and accession number(s) can be found in the article/Supplementary material.

## Supplementary material

Supplementary Figure S1 Epidemiologic trends in childhood CHD mortality across five SDI regions (1990–2021). **(A)** Trends in children aged 5–9; **(B)** trends in children aged 10–14.

Supplementary Figure S2 National burden and EAPC of childhood CHD mortality in 204 countries.

Supplementary Table S1 Mortality from congenital heart disease in children under 5 years between 1990 and 2021 at the global and regional level.

Supplementary Table S2 Mortality from congenital heart disease in children aged 5–9 years between 1990 and 2021 at the global and regional level.

Supplementary Table S3 Mortality from congenital heart disease in children aged 10–14 years between 1990 and 2021 at the global and regional level.

Supplementary Table S4 Mortality of congenital heart disease in children aged 0–14 years between 1990 and 2021 at the national level.

## References

[1] Sun J Mao B Wu Z Jiao X Wang Y Lu Y. Relationship between maternal exposure to heavy metal titanium and offspring congenital heart defects in Lanzhou, China: a nested case-control study. Front Public Health. (2022) 10:946439. 10.3389/fpubh.2022.946439 35991008 PMC9381958

[2] Sevim Bayrak C Zhang P Tristani-Firouzi M Gelb BD Itan Y. *De novo* variants in exomes of congenital heart disease patients identify risk genes and pathways. Genome Med. (2020) 12:9. 10.1186/s13073-019-0709-8 31941532 PMC6961332

[3] Nöthe-Menchen T Wallmeier J Pennekamp P Höben IM Olbrich H Loges NT. Randomization of left-right asymmetry and congenital heart defects: the role of DNAH5 in humans and mice. Circ Genom Precis Med. (2019) 22. 10.1161/CIRCGEN.119.002686 31638833 PMC7174103

[4] Zheng SQ Chen HX Liu XC Yang Q He GW. Genetic analysis of the CITED2 gene promoter in isolated and sporadic congenital ventricular septal defects. J Cell Mol Med. (2021) 25:2254–61. 10.1111/jcmm.16218 33439552 PMC7882930

[5] Khairy P Ionescu-Ittu R Mackie AS Abrahamowicz M Pilote L Marelli AJ. Changing mortality in congenital heart disease. J Am Coll Cardiol. (2010) 56:1149–57. 10.1016/j.jacc.2010.03.085 20863956

[6] Lopez KN Morris SA Sexson Tejtel SK Espaillat A Salemi JL. US mortality attributable to congenital heart disease across the lifespan from 1999 through 2017 exposes persistent racial/ethnic disparities. Circulation. (2020) 142:1132–47. 10.1161/CIRCULATIONAHA.120.046822 32795094 PMC7508797

[7] Roth GA Mensah GA Johnson CO Addolorato G Ammirati E Baddour LM. Global burden of cardiovascular diseases and risk factors, 1990-2019: update from the GBD 2019 study. J Am Coll Cardiol. (2020) 76:2982–3021. 10.1016/j.jacc.2020.11.010 33309175 PMC7755038

[8] Raissadati A Nieminen H Haukka J Sairanen H Jokinen E. Late causes of death after pediatric cardiac surgery: a 60-year population-based study. J Am Coll Cardiol. (2016) 68:487–98. 10.1016/j.jacc.2016.05.038 27470457

[9] Mandalenakis Z Rosengren A Skoglund K Lappas G Eriksson P Dellborg M. Survivorship in children and young adults with congenital heart disease in Sweden. JAMA Intern Med. (2017) 177:224–30. 10.1001/jamainternmed.2016.7765 27992621

[10] Moons P Bovijn L Budts W Belmans A Gewillig M. Temporal trends in survival to adulthood among patients born with congenital heart disease from 1970 to 1992 in Belgium. Circulation. (2010) 122:2264–72. 10.1161/CIRCULATIONAHA.110.946343 21098444

[11] Hofferberth SC Saeed MY Tomholt L Fernandes MC Payne CJ Price K. A geometrically adaptable heart valve replacement. Sci Transl Med. (2020) 12:eaay4006. 10.1126/scitranslmed.aay4006 32075944 PMC7425635

[12] Burchill LJ Gao L Kovacs AH Opotowsky AR Maxwell BG Minnier J. Hospitalization trends and health resource use for adult congenital heart disease-related heart failure. J Am Heart Assoc. (2018) 7:e008775. 10.1161/JAHA.118.008775 30371225 PMC6201452

[13] Global regional and and national burden of congenital heart disease 1990-2017: 1990-2017: a systematic analysis for the global burden of disease study 2017. Lancet Child Adolesc Health. (2020) 4:185–200. 10.1016/S2352-4642(19)30402-X 31978374 PMC7645774

[14] Hamzah M Othman HF Baloglu O Aly H. Outcomes of hypoplastic left heart syndrome: analysis of national inpatient sample database 1998-2004 versus 2005-2014. Eur J Pediatr. (2020) 179:309–16. 10.1007/s00431-019-03508-3 31741094

[15] Radicioni AF De Marco E Gianfrilli D Granato S Gandini L Isidori AM. Strategies and advantages of early diagnosis in Klinefelter's syndrome. Mol Hum Reprod. (2010) 16:434–40. 10.1093/molehr/gaq027 20392711

[16] Su Z Zou Z Hay SI Liu Y Li S Chen H. Global, regional, and national time trends in mortality for congenital heart disease, 1990-2019: an age-period-cohort analysis for the global burden of disease 2019 study. EClinicalMedicine. (2022) 43:101249. 10.1016/j.eclinm.2021.101249 35059612 PMC8760503

[17] Camargo MC Anderson WF King JB Correa P Thomas CC Rosenberg PS. Divergent trends for gastric cancer incidence by anatomical subsite in US adults. Gut. (2011) 60:1644–9. 10.1136/gut.2010.236737 21613644 PMC3202077

[18] Caterino JM Adler D Durham DD Yeung SJ Hudson MF Bastani A. Analysis of diagnoses, symptoms, medications, and admissions among patients with cancer presenting to emergency departments. JAMA Netw Open. (2019) 2:e190979. 10.1001/jamanetworkopen.2019.0979 30901049 PMC6583275

[19] Hu W Fang L Zhang H Ni R Pan G. Changing trends in the air pollution-related disease burden from 1990 to 2019 and its predicted level in 25 years. Environ Sci Pollut Res Int. (2023) 30:1761–73. 10.1007/s11356-022-22318-z 35922595 PMC9362347

[20] Ou Z Yu D Liang Y He W Li Y Zhang M. Analysis of the global burden of disease study highlights the trends in death and disability-adjusted life years of leukemia from 1990 to 2017. Cancer Communi. (2020) 40:598–610. 10.1002/cac2.12094 32936522 PMC7668511

[21] Zhou L Deng Y Li N Zheng Y Tian T Zhai Z. Global, regional, and national burden of Hodgkin lymphoma from 1990 to 2017: estimates from the 2017 global burden of disease study. J Hematol Oncol. (2019) 12:107. 10.1186/s13045-019-0799-1 31640759 PMC6805485

[22] Parikh CR Greenberg JH McArthur E Thiessen-Philbrook H Everett AD Wald R. Incidence of ESKD and mortality among children with congenital heart disease after cardiac surgery. Clin J Am Soc Nephrol. (2019) 14:1450–7. 10.2215/CJN.00690119 31501090 PMC6777584

[23] Ulloa-Cerna AE Jing L Pfeifer JM Raghunath S Ruhl JA Rocha DB. rECHOmmend: an ECG-based machine learning approach for identifying patients at increased risk of undiagnosed structural heart disease detectable by echocardiography. Circulation. (2022) 146:36–47. 10.1161/CIRCULATIONAHA.121.057869 35533093 PMC9241668

[24] Wang S Huang S Gong L Yuan Z Wong J Lee J. Human neonatal thymus mesenchymal stem cells promote neovascularization and cardiac regeneration. Stem Cells Int. (2018) 2018:8503468. 10.1155/2018/8503468 30305821 PMC6165580

[25] van der Bom T Bouma BJ Meijboom FJ Zwinderman AH Mulder BJ. The prevalence of adult congenital heart disease, results from a systematic review and evidence based calculation. Am Heart J. (2012) 164:568–75. 10.1016/j.ahj.2012.07.023 23067916

[26] Wang F Harel-Sterling L Cohen S Liu A Brophy JM Paradis G. Heart failure risk predictions in adult patients with congenital heart disease: a systematic review. Heart. (2019) 105:1661–9. 10.1136/heartjnl-2019-314977 31350277

[27] Ou Y Bloom MS Mai J Francois M Pan W Xiao X. Prenatal detection of congenital heart diseases using echocardiography: 12-year results of an improving program with 9782 cases. Front Public Health. (2022) 10:886262. 10.3389/fpubh.2022.886262 35646777 PMC9136016

[28] Saiyin T Engineer A Greco ER Kim MY Lu X Jones DL. Maternal voluntary exercise mitigates oxidative stress and incidence of congenital heart defects in pre-gestational diabetes. J Cell Mol Med. (2019) 23:5553–65. 10.1111/jcmm.14439 31211496 PMC6653048

[29] Hernández D Millard R Sivakumaran P Wong RC Crombie DE Hewitt AW. Electrical stimulation promotes cardiac differentiation of human induced pluripotent stem cells. Stem Cells Int. (2016) 2016:1718041. 10.1155/2016/1718041 26788064 PMC4691644

[30] Ke B Zeng Y Zhao Z Han F Liu T Wang J. Uric acid: a potent molecular contributor to pluripotent stem cell cardiac differentiation via mesoderm specification. Cell Death Differ. (2019) 26:826–42. 10.1038/s41418-018-0157-9 30038385 PMC6461775

[31] Khalilipalandi S Lemieux A Lauzon-Schnittka J Perreault L Dubois M Tousignant A. Systematic review and meta-analysis of prenatal risk factors for congenital heart disease: part 1, maternal chronic diseases and parental exposures. Can J Cardiol. (2024) 40:2476–95. 10.1016/j.cjca.2024.07.004 38996968

[32] Dai J Wang G Wu C Pan Z Li H Shen L. Exposure to endocrine-disrupting chemicals and congenital heart diseases: the pooled results based on the current evidence. Pediatr Cardiol. (2025) 46:628–38. 10.1007/s00246-024-03478-w 38602518

[33] Li S Wang C Yang C Chen Y Cheng Q Liu J. Prenatal exposure to poly/perfluoroalkyl substances and risk for congenital heart disease in offspring. J Hazard Mater. (2024) 469:134008. 10.1016/j.jhazmat.2024.134008 38503211

