# Supplementary figures and images for: Time trends in mortality of congenital heart disease in children aged 0–14 years: a global, regional, and national cohort analysis from 1990 to 2021 using the global burden of disease study
Source: Front Public Health. 2025 Jul 2;13:1537671. doi: 10.3389/fpubh.2025.1537671 (PMC12263655; doi:10.3389/fpubh.2025.1537671)

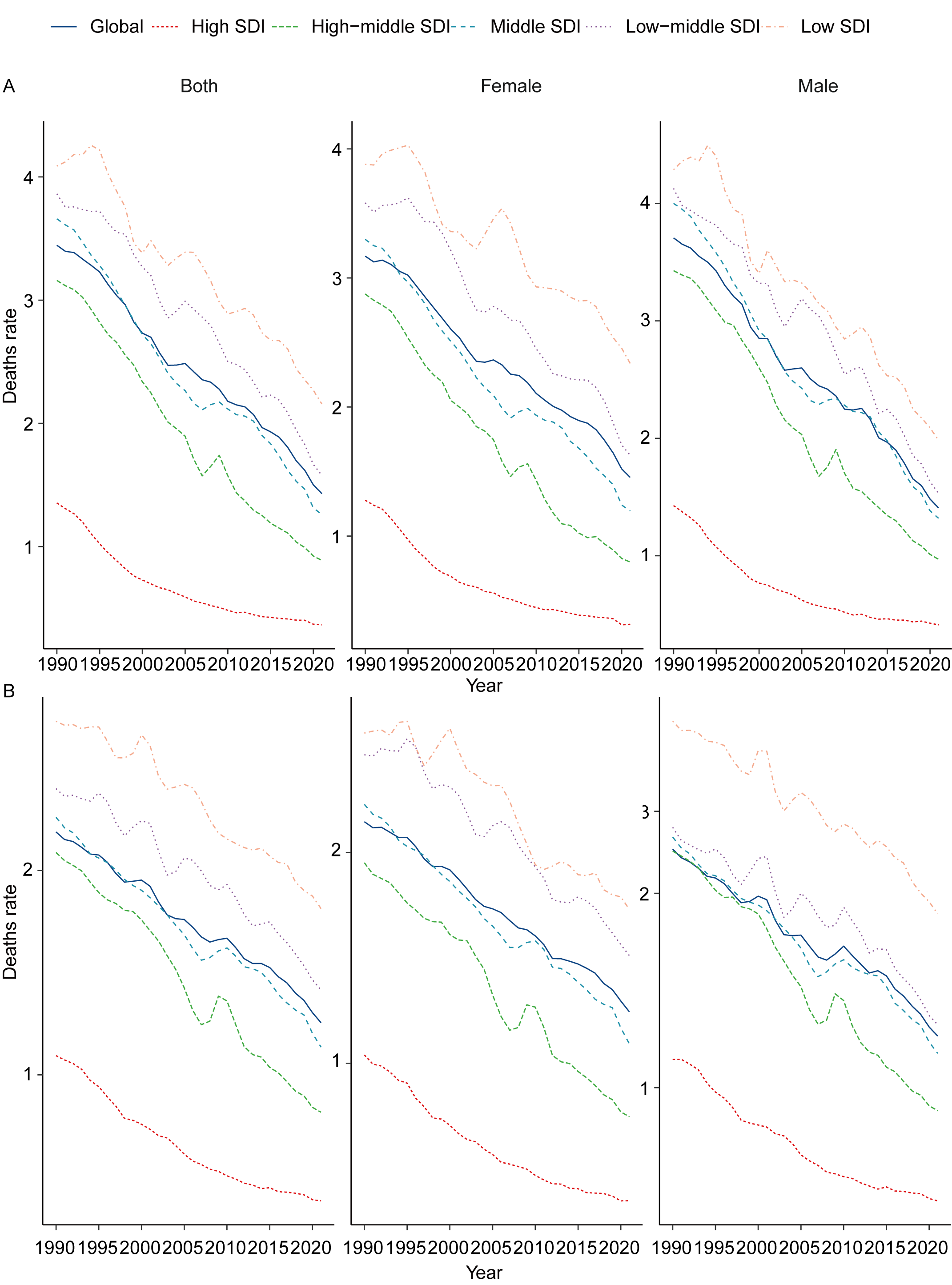

Supplement: Supplementary Figure S1 — Epidemiologic trends in childhood CHD mortality across five SDI regions (1990–2021). (A) Trends in children aged 5–9; (B) trends in children aged 10–14. [file Image_1.tif]

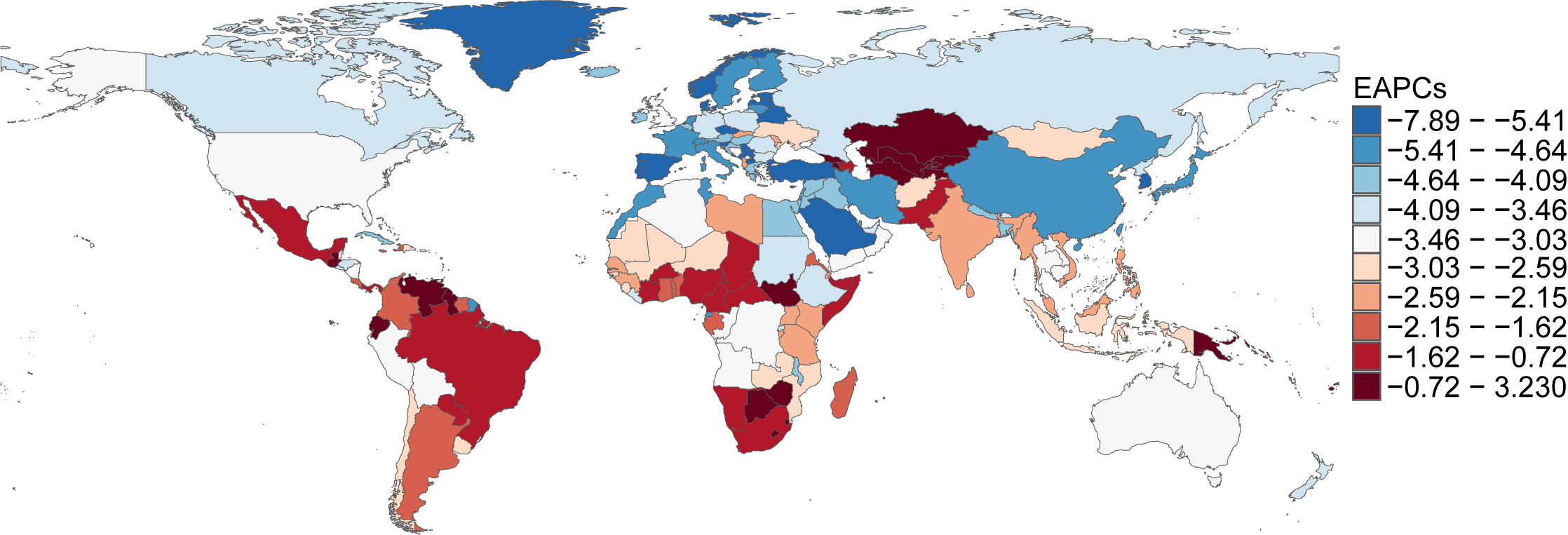

Supplement: Supplementary Figure S2 — National burden and EAPC of childhood CHD mortality in 204 countries. [file Image_2.tif]
