# Supplementary material for: Time trends in mortality of congenital heart disease in children aged 0–14 years: a global, regional, and national cohort analysis from 1990 to 2021 using the global burden of disease study
Source: Front Public Health. 2025 Jul 2;13:1537671. doi: 10.3389/fpubh.2025.1537671 (PMC12263655; doi:10.3389/fpubh.2025.1537671)
Supplement: Supplementary Table S1 — Mortality from congenital heart disease in children under 5 years between 1990 and 2021 at the global and regional level. [file Table_1.docx]

Table S1. Mortality from Congenital Heart Disease in Children Under 5 Years Between 1990 and 2021 at the Global and Regional Level

|  | **1990 (95% UI)** |  |  | **2021 (95% UI)** |  |  |  |  |
| --- | --- | --- | --- | --- | --- | --- | --- | --- |
| **location** | **Deaths Cases** | **Deaths Rate** |  | **Deaths Cases** | **Deaths Rate** |  | **Cases change** | **EAPC** |
| **Global** | 466156.65(261282.55,601016.42) | 75.19(42.15,96.95) |  | 204222.98(165238.47,255409.19) | 31.03(25.11,38.81) |  | -56.19(-66.44,-24.74) | -2.58(-2.70,-2.47) |
| **High SDI** | 17757.79(15242.37,19413.34) | 28.78(24.70,31.46) |  | 3638.40(2918.62,4432.02) | 6.76(5.42,8.23) |  | -79.51(-83.88,-73.45) | -4.34(-4.49,-4.20) |
| **High-middle SDI** | 69371.18(47871.89,86634.26) | 74.67(51.53,93.25) |  | 10284.70(8295.19,12492.79) | 14.68(11.84,17.84) |  | -85.17(-89.20,-76.16) | -5.31(-5.65,-4.97) |
| **Middle SDI** | 150753.58(91645.41,200331.18) | 75.18(45.70,99.90) |  | 41132.42(33692.97,50820.86) | 23.29(19.08,28.77) |  | -72.72(-80.50,-46.75) | -3.38(-3.56,-3.21) |
| **Low-middle SDI** | 142550.18(75050.51,194335.89) | 82.17(43.26,112.02) |  | 68363.08(52761.34,86318.68) | 35.68(27.54,45.06) |  | -52.04(-65.41,5.40) | -2.29(-2.44,-2.14) |
| **Low SDI** | 85339.82(29634.38,125904.18) | 93.99(32.64,138.67) |  | 80576.44(54063.35,109319.61) | 48.66(32.65,66.02) |  | -5.58(-28.53,90.79) | -1.90(-1.99,-1.81) |

Abbreviations: EAPC, estimated annual percentage change; UI, uncertainty interval. EAPC^a^ is expressed as 95% CIs.
