# Supplementary material for: Time trends in mortality of congenital heart disease in children aged 0–14 years: a global, regional, and national cohort analysis from 1990 to 2021 using the global burden of disease study
Source: Front Public Health. 2025 Jul 2;13:1537671. doi: 10.3389/fpubh.2025.1537671 (PMC12263655; doi:10.3389/fpubh.2025.1537671)
Supplement: Supplementary Table S2 — Mortality from congenital heart disease in children aged 5–9 years between 1990 and 2021 at the global and regional level. [file Table_2.docx]

Table S2. Mortality from Congenital Heart Disease in Children Aged 5–9 Years Between 1990 and 2021 at the Global and Regional l Level

|  | **1990 (95% UI)** |  |  | **2021 (95% UI)** |  |  |  |  |
| --- | --- | --- | --- | --- | --- | --- | --- | --- |
| **location** | **Deaths Cases** | **Deaths Rate** |  | **Deaths Cases** | **Deaths Rate** |  | **Cases change** | **EAPC** |
| **Global** | 20102.32(12638.47,25808.87) | 3.44(2.17,4.42) |  | 9824.63(8141.66,12141.51) | 1.43(1.19,1.77) |  | -51.13(-62.42,-16.43) | -2.61(-2.75,-2.46) |
| **High SDI** | 848.00(709.54,941.98) | 1.35(1.13,1.50) |  | 214.33(173.33,262.14) | 0.37(0.30,0.45) |  | -74.72(-79.86,-65.21) | -4.16(-4.46,-3.85) |
| **High-middle SDI** | 2877.09(2162.31,3442.15) | 3.16(2.37,3.78) |  | 731.73(596.29,898.15) | 0.89(0.72,1.09) |  | -74.57(-80.87,-62.24) | -4.15(-4.30,-3.99) |
| **Middle SDI** | 7081.32(4849.21,9221.76) | 3.66(2.51,4.77) |  | 2482.52(2105.79,3059.29) | 1.26(1.07,1.55) |  | -64.94(-73.23,-42.69) | -3.11(-3.31,-2.92) |
| **Low-middle SDI** | 6184.67(3215.40,8612.16) | 3.86(2.01,5.38) |  | 3075.07(2363.64,3970.38) | 1.58(1.21,2.04) |  | -50.28(-65.84,8.61) | -2.63(-2.85,-2.42) |
| **Low SDI** | 3096.19(1285.27,4734.05) | 4.09(1.70,6.25) |  | 3311.10(2504.89,4452.94) | 2.16(1.63,2.90) |  | 6.94(-22.89,125.59) | -2.02(-2.18,-1.86) |

Abbreviations: EAPC, estimated annual percentage change; UI, uncertainty interval. EAPC^a^ is expressed as 95% CIs.
