# Supplementary material for: Time trends in mortality of congenital heart disease in children aged 0–14 years: a global, regional, and national cohort analysis from 1990 to 2021 using the global burden of disease study
Source: Front Public Health. 2025 Jul 2;13:1537671. doi: 10.3389/fpubh.2025.1537671 (PMC12263655; doi:10.3389/fpubh.2025.1537671)
Supplement: Supplementary Table S3 — Mortality from congenital heart disease in children aged 10–14 years between 1990 and 2021 at the global and regional level. [file Table_3.docx]

Table S3. Mortality from Congenital Heart Disease in Children Aged 10–14 Years Between 1990 and 2021 at the Global and Regional l Level

|  | **1990 (95% UI)** |  |  | **2021 (95% UI)** |  |  |  |  |
| --- | --- | --- | --- | --- | --- | --- | --- | --- |
| **location** | **Deaths Cases** | **Deaths Rate** |  | **Deaths Cases** | **Deaths Rate** |  | **Cases change** | **EAPC** |
| **Global** | 11720.23(8561.76,14300.85) | 2.19(1.60,2.67) |  | 8367.58(7122.57,10337.39) | 1.26(1.07,1.55) |  | -28.61(-42.54,3.65) | -1.64(-1.73,-1.56) |
| **High SDI** | 672.23(584.54,723.28) | 1.09(0.95,1.18) |  | 230.00(202.56,279.28) | 0.38(0.34,0.47) |  | -65.79(-70.27,-54.01) | -3.52(-3.67,-3.37) |
| **High-middle SDI** | 1871.70(1486.82,2189.71) | 2.09(1.66,2.44) |  | 640.82(540.86,762.47) | 0.82(0.69,0.97) |  | -65.76(-72.84,-54.57) | -3.03(-3.22,-2.84) |
| **Middle SDI** | 4139.03(3109.39,5169.74) | 2.26(1.70,2.82) |  | 2193.98(1866.98,2590.35) | 1.14(0.97,1.34) |  | -46.99(-56.62,-27.60) | -1.93(-2.05,-1.82) |
| **Low-middle SDI** | 3324.46(2144.33,4220.96) | 2.40(1.55,3.05) |  | 2735.81(2242.86,3535.26) | 1.41(1.16,1.83) |  | -17.71(-39.22,34.54) | -1.58(-1.72,-1.45) |
| **Low SDI** | 1702.58(962.28,2492.37) | 2.73(1.54,4.00) |  | 2559.48(2016.65,3510.90) | 1.81(1.43,2.49) |  | 50.33(14.11,153.69) | -1.32(-1.42,-1.21) |

Abbreviations: EAPC, estimated annual percentage change; UI, uncertainty interval. EAPC^a^ is expressed as 95% CIs.
