# Supplementary material for: Time trends in mortality of congenital heart disease in children aged 0–14 years: a global, regional, and national cohort analysis from 1990 to 2021 using the global burden of disease study
Source: Front Public Health. 2025 Jul 2;13:1537671. doi: 10.3389/fpubh.2025.1537671 (PMC12263655; doi:10.3389/fpubh.2025.1537671)
Supplement: Supplementary Table S4 — Mortality of congenital heart disease in children aged 0–14 years between 1990 and 2021 at the national level. [file Table_4.docx]

Table S4 Mortality of CHD in Children Aged 0–14 Years Between 1990 and 2021at the National Level

| location | 1990 | |  | 2021 | |  | 1990-2021 | |
| --- | --- | --- | --- | --- | --- | --- | --- | --- |
|  | death case | death rate |  | death case | death rate |  | Cases change | EAPC |
| Afghanistan | 6350.21(1475.55,10430.78) | 147.40(34.25,242.12) |  | 8396.39(4115.03,11994.16) | 59.13(28.98,84.46) |  | 32.22(-11.66,221.92) | -2.97(-3.10,-2.85) |
| Albania | 203.19(148.86,278.09) | 18.19(13.32,24.89) |  | 38.26(23.44,56.83) | 8.62(5.28,12.81) |  | -81.17(-89.32,-68.60) | -2.39(-2.50,-2.27) |
| Algeria | 6627.45(3196.39,9352.33) | 61.80(29.80,87.20) |  | 2140.40(1606.93,2777.72) | 16.09(12.08,20.88) |  | -67.70(-78.64,-28.66) | -3.03(-3.46,-2.59) |
| American Samoa | 1.75(1.30,2.30) | 9.19(6.83,12.09) |  | 0.63(0.43,0.90) | 4.45(3.02,6.33) |  | -63.88(-76.51,-44.82) | -2.00(-2.13,-1.88) |
| Andorra | 0.52(0.34,0.69) | 5.47(3.63,7.22) |  | 0.06(0.04,0.09) | 0.63(0.42,0.90) |  | -87.58(-92.53,-77.11) | -6.38(-6.79,-5.97) |
| Angola | 1663.85(394.73,3186.26) | 35.29(8.37,67.58) |  | 1806.99(1127.76,2936.71) | 11.85(7.40,19.26) |  | 8.60(-29.28,239.92) | -3.25(-3.62,-2.88) |
| Antigua and Barbuda | 1.56(1.26,1.91) | 8.56(6.94,10.49) |  | 1.10(0.90,1.30) | 6.50(5.34,7.67) |  | -29.38(-44.03,-12.65) | -0.93(-1.29,-0.57) |
| Argentina | 1488.88(1209.58,1796.34) | 14.69(11.93,17.72) |  | 702.98(557.57,873.94) | 6.90(5.48,8.58) |  | -52.78(-65.26,-37.53) | -1.87(-2.23,-1.51) |
| Armenia | 196.35(148.13,256.21) | 18.82(14.20,24.56) |  | 52.70(39.21,68.95) | 8.90(6.62,11.64) |  | -73.16(-81.71,-62.03) | -0.70(-1.53,0.14) |
| Australia | 197.83(176.16,223.44) | 5.23(4.65,5.90) |  | 77.38(55.21,98.23) | 1.63(1.16,2.07) |  | -60.88(-72.92,-49.08) | -3.18(-3.45,-2.91) |
| Austria | 125.12(99.55,137.91) | 9.28(7.38,10.23) |  | 25.71(20.03,33.09) | 1.98(1.54,2.55) |  | -79.45(-85.14,-69.38) | -4.49(-5.07,-3.91) |
| Azerbaijan | 656.75(481.27,812.33) | 27.06(19.83,33.47) |  | 284.34(174.17,453.85) | 12.05(7.38,19.23) |  | -56.71(-71.59,-32.91) | -1.56(-2.00,-1.11) |
| Bahamas | 8.96(7.27,10.91) | 11.10(9.02,13.53) |  | 3.97(2.85,5.36) | 4.89(3.51,6.60) |  | -55.65(-69.27,-37.09) | -2.63(-2.91,-2.34) |
| Bahrain | 42.17(31.16,54.74) | 25.83(19.09,33.53) |  | 12.34(9.46,15.86) | 4.16(3.19,5.35) |  | -70.74(-79.28,-54.21) | -5.11(-5.60,-4.62) |
| Bangladesh | 19947.83(9674.19,34205.18) | 40.78(19.78,69.93) |  | 4232.73(1999.02,7545.61) | 9.25(4.37,16.49) |  | -78.78(-87.78,-38.74) | -4.37(-4.61,-4.13) |
| Barbados | 7.40(6.09,8.92) | 11.87(9.77,14.30) |  | 3.76(2.66,5.25) | 7.97(5.65,11.15) |  | -49.25(-64.35,-30.46) | -1.08(-1.37,-0.78) |
| Belarus | 429.57(350.89,520.74) | 17.87(14.60,21.66) |  | 29.13(19.68,55.11) | 1.85(1.25,3.49) |  | -93.22(-95.76,-86.46) | -6.88(-7.96,-5.78) |
| Belgium | 134.43(106.16,150.73) | 7.44(5.88,8.35) |  | 41.03(30.81,51.71) | 2.15(1.61,2.70) |  | -69.48(-78.43,-58.45) | -4.16(-4.65,-3.67) |
| Belize | 12.87(10.56,15.82) | 15.73(12.90,19.32) |  | 7.65(6.11,9.67) | 6.21(4.96,7.85) |  | -40.60(-57.43,-19.77) | -2.82(-3.15,-2.49) |
| Benin | 969.23(220.58,1579.09) | 40.02(9.11,65.20) |  | 1288.10(778.91,1942.69) | 21.18(12.81,31.95) |  | 32.90(-11.23,317.98) | -1.64(-1.82,-1.45) |
| Bermuda | 1.02(0.74,1.41) | 8.58(6.23,11.86) |  | 0.23(0.12,0.39) | 2.75(1.38,4.57) |  | -77.34(-85.83,-67.06) | -3.28(-3.59,-2.96) |
| Bhutan | 82.14(31.68,147.44) | 31.33(12.08,56.23) |  | 19.91(9.60,33.92) | 10.64(5.13,18.12) |  | -75.76(-87.27,-30.70) | -3.65(-3.81,-3.50) |
| Bolivia (Plurinational State of) | 1761.34(713.57,2479.66) | 65.58(26.57,92.32) |  | 786.69(581.28,1038.05) | 22.56(16.67,29.77) |  | -55.34(-70.15,-0.39) | -3.06(-3.18,-2.94) |
| Bosnia and Herzegovina | 81.83(54.80,113.81) | 7.47(5.00,10.39) |  | 14.24(10.47,19.53) | 2.90(2.13,3.98) |  | -82.59(-87.62,-73.47) | -3.06(-3.48,-2.64) |
| Botswana | 42.10(28.90,57.25) | 7.13(4.89,9.70) |  | 38.92(24.40,56.74) | 5.57(3.49,8.13) |  | -7.55(-38.17,39.98) | -0.52(-0.66,-0.37) |
| Brazil | 7767.81(6550.01,9066.43) | 14.95(12.61,17.45) |  | 4455.88(3551.14,5446.62) | 9.25(7.37,11.30) |  | -42.64(-57.48,-25.41) | -0.75(-1.15,-0.34) |
| Brunei Darussalam | 11.82(9.10,14.67) | 13.05(10.04,16.20) |  | 6.66(4.97,8.59) | 7.04(5.26,9.09) |  | -43.63(-60.04,-23.50) | -1.68(-1.89,-1.47) |
| Bulgaria | 323.32(282.64,370.17) | 18.62(16.28,21.32) |  | 58.29(47.11,71.15) | 5.97(4.83,7.29) |  | -81.97(-86.66,-77.23) | -3.51(-4.12,-2.91) |
| Burkina Faso | 2207.65(408.24,3612.03) | 46.78(8.65,76.54) |  | 3209.48(1332.30,5016.39) | 30.94(12.84,48.36) |  | 45.38(3.37,258.25) | -1.01(-1.15,-0.88) |
| Burundi | 851.93(222.36,1478.75) | 32.50(8.48,56.42) |  | 731.54(414.22,1189.26) | 12.50(7.08,20.31) |  | -14.13(-43.69,127.19) | -2.22(-2.73,-1.72) |
| Cabo Verde | 33.14(12.43,48.07) | 21.07(7.90,30.55) |  | 6.69(3.88,10.32) | 4.67(2.71,7.21) |  | -79.82(-90.56,-35.80) | -5.01(-5.23,-4.79) |
| Cambodia | 3191.60(854.43,4762.58) | 68.48(18.33,102.18) |  | 1258.34(872.50,1699.58) | 24.59(17.05,33.22) |  | -60.57(-73.96,18.59) | -3.35(-3.40,-3.31) |
| Cameroon | 1518.41(438.59,2375.40) | 31.10(8.98,48.66) |  | 2143.63(1225.09,3012.28) | 15.92(9.10,22.37) |  | 41.18(-4.62,212.84) | -1.60(-1.83,-1.37) |
| Canada | 462.22(377.54,511.26) | 8.04(6.56,8.89) |  | 125.41(99.54,155.36) | 2.03(1.61,2.52) |  | -72.87(-79.05,-63.27) | -3.53(-4.04,-3.02) |
| Central African Republic | 460.63(130.64,880.35) | 37.67(10.68,72.00) |  | 490.19(211.37,852.04) | 21.46(9.26,37.31) |  | 6.42(-24.17,93.88) | -1.50(-1.66,-1.34) |
| Chad | 1013.78(202.73,1615.34) | 34.64(6.93,55.20) |  | 2356.66(810.49,3621.42) | 26.14(8.99,40.17) |  | 132.46(67.13,351.96) | -0.73(-0.80,-0.67) |
| Chile | 418.41(367.70,475.12) | 10.53(9.26,11.96) |  | 116.87(98.31,137.96) | 3.20(2.69,3.78) |  | -72.07(-77.70,-65.30) | -2.76(-3.19,-2.32) |
| China | 114663.43(70812.02,159327.46) | 36.02(22.24,50.04) |  | 14006.11(10616.56,18293.91) | 5.39(4.09,7.05) |  | -87.79(-92.07,-76.47) | -5.37(-5.68,-5.07) |
| Colombia | 2147.41(1783.06,2461.50) | 18.41(15.29,21.11) |  | 1008.76(719.09,1415.79) | 9.50(6.78,13.34) |  | -53.02(-68.24,-34.74) | -1.62(-1.99,-1.25) |
| Comoros | 58.64(18.14,114.55) | 27.57(8.53,53.86) |  | 25.93(15.07,47.92) | 10.80(6.27,19.95) |  | -55.78(-72.60,2.23) | -2.89(-3.08,-2.70) |
| Congo | 195.94(72.49,369.12) | 18.61(6.88,35.06) |  | 129.62(83.24,203.34) | 6.72(4.31,10.54) |  | -33.84(-57.01,67.77) | -3.19(-3.66,-2.71) |
| Cook Islands | 0.42(0.25,0.61) | 6.31(3.73,9.21) |  | 0.07(0.03,0.18) | 1.98(0.85,4.75) |  | -82.01(-93.33,-36.41) | -6.01(-6.80,-5.20) |
| Costa Rica | 157.27(142.36,173.99) | 13.99(12.66,15.48) |  | 66.45(53.73,81.40) | 6.53(5.28,8.00) |  | -57.75(-66.97,-47.29) | -1.75(-2.04,-1.45) |
| Croatia | 1768.17(497.97,2861.70) | 31.00(8.73,50.18) |  | 1990.85(1228.28,2911.18) | 17.20(10.61,25.16) |  | -84.48(-88.42,-78.22) | -1.48(-1.68,-1.28) |
| Cuba | 99.43(80.18,109.58) | 10.07(8.12,11.10) |  | 15.43(11.70,20.34) | 2.58(1.96,3.41) |  | -85.29(-88.29,-79.21) | -4.29(-4.61,-3.97) |
| Cyprus | 418.55(370.37,458.27) | 16.72(14.79,18.30) |  | 61.56(48.56,83.51) | 3.46(2.73,4.70) |  | -83.29(-90.47,-63.41) | -4.40(-4.70,-4.10) |
| Czechia | 18.37(9.22,25.86) | 9.28(4.66,13.07) |  | 3.07(2.14,4.17) | 1.40(0.98,1.90) |  | -90.97(-94.61,-85.65) | -5.64(-5.95,-5.34) |
| C么te d'Ivoire | 184.48(139.16,206.84) | 8.37(6.32,9.39) |  | 16.67(10.56,22.52) | 0.97(0.62,1.31) |  | 12.59(-24.57,199.76) | -6.00(-6.48,-5.51) |
| Democratic People's Republic of Korea | 1714.00(1204.49,2348.82) | 28.81(20.25,39.48) |  | 388.38(243.15,613.07) | 8.14(5.09,12.84) |  | -77.34(-87.12,-59.28) | -3.62(-3.92,-3.33) |
| Democratic Republic of the Congo | 5194.28(1601.65,9255.87) | 29.34(9.05,52.28) |  | 3622.45(2126.38,5954.96) | 9.53(5.60,15.67) |  | -30.26(-54.19,69.81) | -3.07(-3.42,-2.72) |
| Denmark | 118.32(97.29,133.77) | 13.39(11.01,15.14) |  | 21.29(15.57,26.65) | 2.23(1.63,2.79) |  | -82.00(-86.95,-75.14) | -6.07(-6.58,-5.55) |
| Djibouti | 33.67(10.22,68.92) | 19.34(5.87,39.58) |  | 35.73(20.12,65.15) | 8.65(4.87,15.77) |  | 6.12(-37.16,174.99) | -2.29(-2.70,-1.87) |
| Dominica | 2.48(1.90,3.19) | 9.98(7.66,12.84) |  | 1.49(0.97,2.11) | 10.86(7.11,15.45) |  | -40.00(-61.39,-11.37) | 0.51(0.12,0.90) |
| Dominican Republic | 592.16(459.32,838.80) | 21.97(17.04,31.12) |  | 231.24(124.66,444.38) | 7.87(4.24,15.12) |  | -60.95(-78.74,-22.35) | -2.89(-3.11,-2.66) |
| Ecuador | 659.92(548.42,760.24) | 17.07(14.19,19.67) |  | 570.34(429.58,741.81) | 11.25(8.47,14.63) |  | -13.57(-36.80,15.66) | -0.37(-0.68,-0.07) |
| Egypt | 16213.05(5545.42,24557.41) | 73.09(25.00,110.71) |  | 5145.84(3798.63,6807.29) | 13.96(10.31,18.47) |  | -68.26(-82.05,1.91) | -4.19(-4.65,-3.73) |
| El Salvador | 793.15(426.38,1057.74) | 36.75(19.76,49.01) |  | 166.51(105.56,248.02) | 9.16(5.80,13.64) |  | -79.01(-87.56,-47.91) | -3.98(-4.25,-3.71) |
| Equatorial Guinea | 50.93(17.25,99.66) | 25.87(8.76,50.61) |  | 38.82(22.86,62.28) | 6.64(3.91,10.65) |  | -23.79(-60.70,181.82) | -4.68(-4.90,-4.46) |
| Eritrea | 375.38(94.25,761.35) | 23.58(5.92,47.83) |  | 326.06(177.15,632.58) | 12.92(7.02,25.06) |  | -13.14(-47.86,134.54) | -1.63(-1.79,-1.47) |
| Estonia | 41.97(36.51,47.32) | 12.02(10.46,13.55) |  | 2.70(1.63,4.03) | 1.25(0.75,1.86) |  | -93.57(-96.43,-89.47) | -6.72(-7.42,-6.01) |
| Eswatini | 36.41(22.06,50.65) | 9.44(5.72,13.13) |  | 27.03(18.11,39.63) | 6.55(4.39,9.61) |  | -25.76(-51.65,46.55) | -0.60(-0.91,-0.29) |
| Ethiopia | 8585.27(2054.94,17627.61) | 35.24(8.43,72.35) |  | 5314.02(3108.48,9536.25) | 11.98(7.01,21.50) |  | -38.10(-61.34,74.96) | -3.67(-3.81,-3.53) |
| Fiji | 54.23(41.30,68.31) | 19.27(14.67,24.27) |  | 47.90(34.13,64.92) | 17.58(12.52,23.82) |  | -11.67(-39.92,31.55) | -0.04(-0.36,0.27) |
| Finland | 82.83(71.30,93.51) | 8.58(7.39,9.69) |  | 14.63(9.99,19.42) | 1.73(1.18,2.29) |  | -82.34(-87.26,-75.57) | -5.31(-5.55,-5.07) |
| France | 907.28(745.20,971.22) | 7.75(6.36,8.29) |  | 189.21(142.22,268.58) | 1.63(1.23,2.31) |  | -79.14(-84.93,-66.75) | -4.66(-4.91,-4.41) |
| Gabon | 60.15(29.32,113.11) | 14.76(7.20,27.76) |  | 37.97(24.10,64.83) | 5.94(3.77,10.14) |  | -36.88(-61.87,47.83) | -2.11(-2.57,-1.65) |
| Gambia | 109.02(31.34,165.76) | 23.63(6.79,35.93) |  | 106.27(68.60,150.17) | 10.70(6.91,15.12) |  | -2.52(-41.41,164.93) | -2.62(-2.89,-2.36) |
| Georgia | 117.75(87.88,146.29) | 8.60(6.42,10.69) |  | 49.81(36.20,65.17) | 6.77(4.92,8.85) |  | -57.70(-68.35,-42.77) | 1.01(0.28,1.75) |
| Germany | 1116.76(941.07,1296.45) | 8.63(7.27,10.01) |  | 277.53(210.53,332.65) | 2.32(1.76,2.78) |  | -75.15(-81.24,-68.74) | -3.86(-4.28,-3.44) |
| Ghana | 1446.90(454.57,2256.19) | 21.54(6.77,33.59) |  | 1270.70(785.89,1778.22) | 9.86(6.10,13.80) |  | -12.18(-46.31,156.49) | -1.83(-2.05,-1.61) |
| Greece | 263.18(220.63,294.11) | 13.01(10.90,14.53) |  | 43.02(34.10,52.91) | 3.08(2.44,3.79) |  | -83.65(-87.66,-77.18) | -4.41(-4.74,-4.09) |
| Greenland | 2.15(1.14,3.18) | 15.10(8.04,22.37) |  | 0.33(0.21,0.57) | 2.80(1.81,4.85) |  | -84.68(-91.91,-68.90) | -5.50(-5.88,-5.11) |
| Grenada | 5.23(4.24,6.46) | 15.67(12.69,19.33) |  | 2.16(1.70,2.74) | 9.90(7.79,12.53) |  | -58.72(-67.45,-46.07) | -0.94(-1.19,-0.68) |
| Guam | 3.49(2.80,4.54) | 8.36(6.71,10.88) |  | 2.09(1.51,2.90) | 5.71(4.14,7.93) |  | -40.07(-57.20,-20.08) | -0.28(-0.73,0.18) |
| Guatemala | 270.84(223.09,345.25) | 6.67(5.49,8.50) |  | 550.02(394.12,723.14) | 11.15(7.99,14.66) |  | 103.08(42.06,186.74) | 3.23(2.43,4.05) |
| Guinea | 1497.89(299.57,2590.00) | 54.43(10.89,94.12) |  | 1428.72(809.26,2110.96) | 23.63(13.39,34.92) |  | -4.62(-37.37,200.09) | -2.17(-2.36,-1.98) |
| Guinea-Bissau | 199.58(38.33,327.64) | 41.37(7.94,67.92) |  | 135.53(82.41,192.89) | 15.09(9.18,21.48) |  | -32.10(-59.56,140.96) | -2.84(-3.21,-2.47) |
| Guyana | 49.61(40.64,60.31) | 16.88(13.82,20.52) |  | 23.27(16.45,31.53) | 10.90(7.71,14.78) |  | -53.09(-67.66,-33.21) | -0.57(-0.85,-0.29) |
| Haiti | 2292.30(1460.90,3233.53) | 84.49(53.85,119.18) |  | 1868.69(1027.49,3141.80) | 42.93(23.61,72.18) |  | -18.48(-44.36,41.02) | -1.72(-2.00,-1.43) |
| Honduras | 671.60(387.57,946.32) | 30.40(17.54,42.83) |  | 301.00(207.10,444.81) | 9.18(6.32,13.57) |  | -55.18(-72.38,-4.35) | -3.68(-3.75,-3.61) |
| Hungary | 256.57(212.33,295.46) | 12.04(9.96,13.86) |  | 36.46(26.04,46.80) | 2.63(1.88,3.37) |  | -85.79(-90.53,-80.16) | -4.58(-4.86,-4.29) |
| Iceland | 4.72(3.86,5.38) | 7.44(6.09,8.47) |  | 1.32(0.96,1.71) | 1.95(1.43,2.54) |  | -72.05(-79.10,-60.44) | -4.43(-4.81,-4.04) |
| India | 77025.09(45660.64,104034.19) | 23.59(13.98,31.86) |  | 35232.29(25272.40,50611.22) | 9.62(6.90,13.81) |  | -54.26(-69.65,6.14) | -2.50(-2.67,-2.33) |
| Indonesia | 15197.44(8075.13,20700.04) | 22.44(11.92,30.56) |  | 6978.23(4920.53,9432.48) | 10.37(7.31,14.02) |  | -54.08(-69.71,7.94) | -2.59(-2.74,-2.45) |
| Iran (Islamic Republic of) | 13351.78(7178.14,18165.80) | 52.60(28.28,71.56) |  | 822.56(574.84,1147.36) | 4.08(2.85,5.69) |  | -93.84(-96.63,-85.62) | -4.85(-6.00,-3.69) |
| Iraq | 4585.78(2435.65,6285.90) | 55.68(29.57,76.32) |  | 1804.72(1261.23,2553.70) | 13.41(9.37,18.97) |  | -60.65(-76.94,-4.95) | -4.35(-4.69,-4.01) |
| Ireland | 63.72(54.53,69.60) | 6.48(5.55,7.08) |  | 16.63(12.72,21.18) | 1.67(1.28,2.12) |  | -73.90(-79.89,-64.62) | -4.13(-4.58,-3.68) |
| Israel | 150.02(119.03,173.67) | 9.79(7.76,11.33) |  | 51.33(38.34,64.51) | 1.95(1.46,2.46) |  | -65.78(-76.22,-51.66) | -4.42(-4.67,-4.17) |
| Italy | 769.11(657.89,863.80) | 8.33(7.13,9.36) |  | 143.88(105.83,176.87) | 1.89(1.39,2.33) |  | -81.29(-87.38,-76.15) | -5.11(-5.29,-4.92) |
| Jamaica | 106.01(79.61,131.07) | 12.69(9.53,15.69) |  | 37.64(27.18,51.03) | 6.45(4.65,8.74) |  | -64.49(-76.00,-45.98) | -1.68(-2.07,-1.29) |
| Japan | 1828.21(1613.20,1988.91) | 7.92(6.99,8.61) |  | 267.52(183.60,363.99) | 1.73(1.19,2.36) |  | -85.37(-89.82,-78.74) | -4.64(-4.85,-4.43) |
| Jordan | 645.94(446.18,814.17) | 39.55(27.32,49.85) |  | 362.38(280.22,489.31) | 9.97(7.71,13.47) |  | -43.90(-62.07,0.90) | -4.34(-4.56,-4.12) |
| Kazakhstan | 835.12(630.25,1057.55) | 16.07(12.13,20.35) |  | 637.37(507.13,805.70) | 11.75(9.35,14.85) |  | -23.68(-43.95,5.66) | -0.28(-1.32,0.76) |
| Kenya | 1383.84(575.73,2945.55) | 12.39(5.15,26.37) |  | 938.87(515.73,1856.78) | 5.03(2.76,9.95) |  | -32.16(-59.33,59.12) | -2.25(-2.55,-1.95) |
| Kiribati | 11.65(2.94,18.25) | 39.43(9.94,61.79) |  | 7.64(3.15,11.21) | 18.19(7.50,26.67) |  | -34.38(-51.28,14.04) | -2.29(-2.39,-2.18) |
| Kuwait | 122.95(101.14,152.47) | 22.18(18.24,27.50) |  | 60.06(48.82,74.88) | 7.10(5.77,8.86) |  | -51.15(-64.82,-32.82) | -3.29(-4.01,-2.57) |
| Kyrgyzstan | 260.65(212.21,304.32) | 15.54(12.65,18.14) |  | 268.66(215.38,327.36) | 11.81(9.47,14.39) |  | 3.07(-24.95,35.37) | 0.50(0.01,1.00) |
| Lao People's Democratic Republic | 1462.29(327.56,2247.38) | 79.34(17.77,121.93) |  | 736.07(441.98,1066.33) | 32.05(19.25,46.44) |  | -49.66(-66.15,57.36) | -3.07(-3.18,-2.97) |
| Latvia | 103.10(90.62,122.45) | 18.12(15.93,21.52) |  | 5.51(4.18,7.80) | 1.85(1.41,2.63) |  | -94.66(-96.40,-91.90) | -5.93(-6.71,-5.14) |
| Lebanon | 219.82(96.58,327.34) | 21.02(9.23,31.30) |  | 55.11(37.06,83.19) | 4.31(2.90,6.51) |  | -74.93(-85.56,-42.03) | -4.94(-5.26,-4.61) |
| Lesotho | 68.40(37.10,105.33) | 10.02(5.43,15.43) |  | 52.16(29.56,82.46) | 8.27(4.69,13.08) |  | -23.74(-49.91,22.34) | -0.56(-0.68,-0.43) |
| Liberia | 666.43(112.36,1165.28) | 58.97(9.94,103.11) |  | 343.45(197.86,519.40) | 15.71(9.05,23.76) |  | -48.46(-66.42,98.20) | -3.78(-4.08,-3.47) |
| Libya | 770.65(483.18,1040.63) | 42.56(26.68,57.46) |  | 262.73(175.51,371.77) | 17.61(11.77,24.92) |  | -65.91(-76.16,-48.45) | -2.15(-2.60,-1.69) |
| Lithuania | 127.34(113.76,148.77) | 15.33(13.69,17.91) |  | 11.50(8.33,15.16) | 2.82(2.04,3.72) |  | -90.97(-94.11,-87.85) | -4.70(-5.08,-4.33) |
| Luxembourg | 3.67(3.08,4.24) | 5.55(4.66,6.42) |  | 1.06(0.72,1.60) | 1.05(0.71,1.58) |  | -71.05(-80.42,-54.64) | -5.45(-6.33,-4.55) |
| Madagascar | 1367.91(374.37,2792.14) | 25.07(6.86,51.18) |  | 1285.31(694.55,2276.93) | 10.95(5.92,19.41) |  | -6.04(-37.67,106.38) | -2.14(-2.36,-1.92) |
| Malawi | 1905.32(424.91,3718.60) | 41.88(9.34,81.73) |  | 869.99(517.41,1468.10) | 10.71(6.37,18.07) |  | -54.34(-73.12,64.71) | -4.11(-4.27,-3.95) |
| Malaysia | 719.11(436.82,950.61) | 10.94(6.65,14.46) |  | 311.83(234.28,401.48) | 4.10(3.08,5.27) |  | -56.64(-70.31,-16.36) | -2.49(-3.16,-1.82) |
| Maldives | 32.14(9.60,48.33) | 30.60(9.14,46.01) |  | 6.90(4.95,9.69) | 6.88(4.95,9.68) |  | -78.54(-88.50,-16.70) | -3.81(-4.25,-3.37) |
| Mali | 2872.08(1407.21,4863.49) | 69.54(34.07,117.77) |  | 3332.11(1912.78,4932.08) | 28.79(16.52,42.61) |  | 16.02(-27.10,189.39) | -2.64(-2.80,-2.48) |
| Malta | 8.11(6.16,9.30) | 9.27(7.04,10.62) |  | 2.59(2.01,3.30) | 4.04(3.13,5.15) |  | -68.12(-75.90,-58.22) | -1.88(-2.53,-1.23) |
| Marshall Islands | 2.98(1.55,4.03) | 13.56(7.08,18.38) |  | 1.76(1.18,2.56) | 10.06(6.73,14.66) |  | -40.99(-62.04,-3.73) | -1.01(-1.52,-0.51) |
| Mauritania | 196.96(52.30,294.31) | 21.31(5.66,31.84) |  | 156.53(106.65,220.17) | 8.45(5.76,11.88) |  | -20.53(-50.62,136.31) | -2.81(-3.22,-2.41) |
| Mauritius | 43.59(37.35,55.60) | 13.21(11.32,16.85) |  | 19.50(15.59,26.13) | 9.40(7.52,12.60) |  | -55.26(-64.19,-44.74) | -1.88(-2.26,-1.49) |
| Mexico | 5758.78(5016.84,6895.04) | 17.23(15.01,20.63) |  | 4183.38(3132.45,5401.94) | 13.05(9.77,16.85) |  | -27.36(-47.66,-1.02) | -0.75(-1.11,-0.39) |
| Micronesia (Federated States of) | 9.15(3.87,13.03) | 19.93(8.42,28.36) |  | 2.31(1.69,3.19) | 7.55(5.51,10.43) |  | -74.75(-83.83,-45.07) | -3.12(-3.20,-3.03) |
| Monaco | 0.24(0.17,0.33) | 6.94(4.93,9.46) |  | 0.15(0.10,0.21) | 2.94(2.04,4.23) |  | -40.11(-62.55,-5.21) | -4.09(-4.71,-3.47) |
| Mongolia | 262.90(116.46,384.85) | 29.21(12.94,42.76) |  | 103.24(71.19,136.87) | 9.50(6.55,12.60) |  | -60.73(-78.19,-0.19) | -2.67(-3.14,-2.19) |
| Montenegro | 12.80(9.15,17.34) | 7.92(5.66,10.73) |  | 1.54(0.97,2.49) | 1.38(0.87,2.24) |  | -87.99(-92.87,-76.51) | -5.57(-6.08,-5.05) |
| Morocco | 2765.38(1921.23,3715.62) | 28.26(19.63,37.97) |  | 564.08(320.95,1182.44) | 5.76(3.28,12.08) |  | -79.60(-89.15,-56.82) | -4.66(-5.04,-4.28) |
| Mozambique | 2734.98(617.19,5282.05) | 44.08(9.95,85.14) |  | 2259.24(1246.48,4061.09) | 15.84(8.74,28.47) |  | -17.39(-47.73,129.11) | -2.95(-3.13,-2.77) |
| Myanmar | 9533.29(2731.69,15238.68) | 64.52(18.49,103.13) |  | 4813.49(2882.51,6775.56) | 30.83(18.46,43.39) |  | -49.51(-66.40,26.66) | -2.55(-2.83,-2.26) |
| Namibia | 51.06(31.45,71.43) | 8.50(5.24,11.89) |  | 42.26(27.22,62.97) | 5.12(3.30,7.63) |  | -17.24(-49.07,57.58) | -0.95(-1.26,-0.64) |
| Nauru | 0.87(0.44,1.23) | 20.51(10.37,29.13) |  | 0.59(0.37,0.87) | 14.89(9.34,21.77) |  | -31.55(-52.93,4.62) | -1.04(-1.72,-0.36) |
| Nepal | 1854.72(1289.12,2520.13) | 22.01(15.30,29.91) |  | 520.88(278.31,1147.63) | 5.65(3.02,12.44) |  | -71.92(-85.93,-33.71) | -4.38(-4.43,-4.32) |
| Netherlands | 225.45(185.90,251.69) | 8.27(6.82,9.24) |  | 48.15(39.45,62.72) | 1.80(1.47,2.34) |  | -78.64(-83.19,-69.21) | -5.26(-5.57,-4.95) |
| New Zealand | 49.53(43.81,55.94) | 6.19(5.47,6.99) |  | 17.18(12.70,23.50) | 1.75(1.29,2.39) |  | -65.31(-73.87,-54.53) | -4.06(-4.46,-3.65) |
| Nicaragua | 549.86(281.35,798.49) | 30.19(15.45,43.84) |  | 161.54(107.76,235.21) | 8.16(5.44,11.88) |  | -70.62(-83.99,-29.35) | -3.45(-3.72,-3.17) |
| Niger | 1945.91(281.22,3345.56) | 47.89(6.92,82.34) |  | 2630.73(1104.57,4072.81) | 20.61(8.65,31.91) |  | 35.19(-7.63,333.34) | -2.92(-3.15,-2.69) |
| Nigeria | 13433.19(2953.73,20765.65) | 34.33(7.55,53.08) |  | 21622.00(10881.15,33068.64) | 21.28(10.71,32.55) |  | 60.96(18.33,297.60) | -1.08(-1.29,-0.87) |
| Niue | 0.10(0.07,0.14) | 12.45(8.29,16.77) |  | 0.13(0.10,0.16) | 33.76(26.55,42.22) |  | 30.15(-3.29,84.86) | 0.59(-0.36,1.55) |
| North Macedonia | 129.73(93.24,163.46) | 24.63(17.70,31.03) |  | 9.03(6.49,12.90) | 2.76(1.98,3.94) |  | -93.04(-95.59,-86.99) | -5.59(-6.13,-5.04) |
| Northern Mariana Islands | 0.76(0.55,1.09) | 6.29(4.55,8.95) |  | 0.31(0.22,0.42) | 2.72(1.97,3.72) |  | -59.98(-69.50,-44.41) | -2.32(-2.71,-1.92) |
| Norway | 67.01(61.47,74.37) | 8.39(7.70,9.32) |  | 11.61(7.54,19.98) | 1.26(0.82,2.16) |  | -82.67(-88.83,-70.13) | -5.91(-6.22,-5.60) |
| Oman | 246.61(142.69,353.38) | 29.35(16.98,42.05) |  | 76.24(58.26,102.65) | 6.23(4.76,8.39) |  | -69.09(-80.97,-35.30) | -3.27(-4.32,-2.22) |
| Pakistan | 11659.62(7946.30,15638.93) | 23.68(16.14,31.76) |  | 11306.14(6732.04,16758.06) | 13.23(7.88,19.61) |  | -3.03(-34.08,67.06) | -0.98(-1.36,-0.59) |
| Palau | 0.73(0.39,1.07) | 15.95(8.50,23.54) |  | 0.24(0.17,0.32) | 7.30(5.22,9.82) |  | -67.24(-77.80,-42.46) | -2.12(-2.39,-1.85) |
| Palestine | 438.87(277.86,593.60) | 45.32(28.70,61.30) |  | 170.86(122.05,239.03) | 9.15(6.54,12.80) |  | -61.07(-76.80,-28.41) | -4.71(-5.00,-4.42) |
| Panama | 192.14(161.57,230.82) | 23.04(19.38,27.68) |  | 170.42(129.12,217.32) | 14.78(11.20,18.84) |  | -11.30(-34.52,22.42) | -1.09(-1.29,-0.89) |
| Papua New Guinea | 763.27(206.00,1188.42) | 44.90(12.12,69.90) |  | 1457.37(530.72,2249.80) | 37.21(13.55,57.44) |  | 90.94(43.00,191.36) | -0.43(-0.56,-0.29) |
| Paraguay | 282.22(207.43,405.33) | 16.90(12.42,24.28) |  | 197.13(123.77,295.31) | 9.82(6.16,14.71) |  | -30.15(-63.04,26.69) | -1.28(-1.50,-1.06) |
| Peru | 2668.61(1395.52,3586.46) | 32.15(16.81,43.21) |  | 840.26(524.58,1207.46) | 8.81(5.50,12.66) |  | -68.51(-82.43,-17.63) | -3.34(-3.73,-2.96) |
| Philippines | 6978.66(4098.48,9801.58) | 27.68(16.25,38.87) |  | 4137.03(3240.71,5516.14) | 12.17(9.53,16.22) |  | -40.72(-57.60,6.54) | -2.15(-2.33,-1.97) |
| Poland | 1567.70(1298.22,1808.16) | 16.37(13.56,18.88) |  | 230.46(171.69,287.07) | 3.92(2.92,4.88) |  | -85.30(-90.30,-80.77) | -3.98(-4.45,-3.52) |
| Portugal | 239.53(199.13,276.39) | 11.32(9.41,13.06) |  | 27.69(20.24,34.46) | 2.03(1.49,2.53) |  | -88.44(-92.21,-84.65) | -6.21(-6.62,-5.80) |
| Puerto Rico | 88.66(76.34,99.79) | 8.90(7.67,10.02) |  | 14.73(11.21,18.18) | 3.31(2.52,4.09) |  | -83.39(-87.12,-78.96) | -3.48(-3.83,-3.13) |
| Qatar | 26.84(16.54,37.40) | 21.46(13.23,29.91) |  | 18.25(12.39,25.52) | 3.69(2.51,5.17) |  | -32.02(-57.36,31.49) | -4.83(-5.10,-4.55) |
| Republic of Korea | 1418.31(879.21,1853.09) | 12.47(7.73,16.30) |  | 81.79(59.01,120.46) | 1.35(0.97,1.98) |  | -94.23(-96.58,-87.92) | -6.65(-6.96,-6.33) |
| Republic of Moldova | 261.13(186.29,344.92) | 21.13(15.07,27.91) |  | 36.11(26.19,48.52) | 6.91(5.01,9.29) |  | -86.17(-90.29,-79.88) | -2.55(-2.98,-2.11) |
| Romania | 1100.04(915.66,1314.41) | 19.76(16.44,23.61) |  | 159.78(132.72,189.72) | 5.31(4.41,6.30) |  | -85.48(-89.33,-81.55) | -3.65(-4.10,-3.20) |
| Russian Federation | 4290.18(3762.43,5360.14) | 12.36(10.84,15.45) |  | 789.66(606.46,1024.60) | 3.03(2.33,3.93) |  | -81.59(-87.60,-74.63) | -3.46(-4.50,-2.40) |
| Rwanda | 1039.10(269.09,1970.68) | 30.63(7.93,58.08) |  | 532.77(321.65,875.06) | 10.72(6.47,17.61) |  | -48.73(-71.15,49.13) | -3.64(-4.02,-3.26) |
| Saint Kitts and Nevis | 1.97(1.68,2.29) | 13.93(11.88,16.24) |  | 0.78(0.59,1.02) | 7.90(5.96,10.31) |  | -60.44(-70.38,-47.21) | -1.68(-1.84,-1.53) |
| Saint Lucia | 6.22(4.94,7.52) | 12.06(9.59,14.59) |  | 1.90(1.35,2.62) | 6.41(4.54,8.84) |  | -69.37(-79.43,-55.50) | -1.57(-1.86,-1.27) |
| Saint Vincent and the Grenadines | 6.02(4.82,7.42) | 14.64(11.73,18.06) |  | 1.46(1.09,1.92) | 5.85(4.38,7.71) |  | -75.72(-82.96,-66.04) | -2.70(-3.05,-2.36) |
| Samoa | 11.34(6.52,16.06) | 15.91(9.14,22.54) |  | 6.33(4.28,8.94) | 7.92(5.35,11.18) |  | -44.19(-66.65,9.00) | -2.04(-2.11,-1.97) |
| San Marino | 0.12(0.08,0.17) | 2.87(1.94,4.18) |  | 0.02(0.01,0.03) | 0.45(0.25,0.80) |  | -83.30(-91.44,-65.15) | -5.80(-6.04,-5.56) |
| Sao Tome and Principe | 15.28(4.45,23.32) | 26.96(7.85,41.15) |  | 4.49(2.63,7.50) | 5.77(3.38,9.63) |  | -70.62(-86.87,31.89) | -4.63(-5.03,-4.24) |
| Saudi Arabia | 2566.45(1334.98,3715.73) | 39.16(20.37,56.70) |  | 219.14(124.66,365.89) | 2.90(1.65,4.84) |  | -91.46(-96.14,-77.07) | -7.89(-8.03,-7.75) |
| Senegal | 1175.96(289.76,1852.46) | 32.21(7.94,50.73) |  | 791.88(555.17,1096.09) | 12.45(8.73,17.23) |  | -32.66(-59.47,132.19) | -2.55(-2.93,-2.17) |
| Serbia | 531.08(335.31,689.57) | 24.49(15.46,31.79) |  | 37.94(27.87,52.05) | 2.86(2.10,3.92) |  | -92.86(-95.46,-86.87) | -7.44(-8.07,-6.81) |
| Seychelles | 3.18(2.58,3.96) | 13.41(10.88,16.71) |  | 2.65(1.94,3.51) | 11.34(8.27,14.99) |  | -16.55(-39.22,15.00) | 0.28(-0.02,0.57) |
| Sierra Leone | 1169.64(203.32,1984.71) | 64.53(11.22,109.49) |  | 958.65(483.37,1475.52) | 26.81(13.52,41.26) |  | -18.04(-47.99,167.72) | -2.73(-2.84,-2.62) |
| Singapore | 103.37(87.48,114.61) | 15.92(13.47,17.65) |  | 11.60(7.33,17.48) | 1.43(0.90,2.15) |  | -88.78(-92.86,-81.58) | -6.73(-7.42,-6.03) |
| Slovakia | 144.69(118.24,168.68) | 10.91(8.92,12.72) |  | 34.63(26.65,45.19) | 4.04(3.11,5.28) |  | -76.07(-82.46,-68.23) | -2.55(-2.93,-2.16) |
| Slovenia | 36.03(27.58,40.90) | 8.71(6.67,9.89) |  | 3.98(2.70,5.11) | 1.27(0.86,1.63) |  | -88.96(-92.91,-82.97) | -5.57(-5.78,-5.35) |
| Solomon Islands | 39.59(19.18,56.75) | 25.43(12.32,36.45) |  | 34.96(24.64,47.59) | 13.44(9.47,18.30) |  | -11.70(-39.75,70.82) | -2.03(-2.20,-1.86) |
| Somalia | 1136.49(253.55,2457.10) | 29.17(6.51,63.07) |  | 1844.75(655.07,3924.65) | 17.86(6.34,37.99) |  | 62.32(12.38,186.72) | -1.06(-1.32,-0.80) |
| South Africa | 1235.10(1003.90,1645.36) | 9.07(7.37,12.09) |  | 831.87(553.50,1202.57) | 5.47(3.64,7.91) |  | -32.65(-56.55,5.04) | -0.99(-1.19,-0.80) |
| South Sudan | 901.98(198.37,1851.93) | 34.37(7.56,70.57) |  | 1223.76(436.56,2189.75) | 28.49(10.16,50.98) |  | 35.67(-9.43,149.50) | -0.35(-0.70,-0.01) |
| Spain | 667.41(576.24,753.65) | 8.52(7.35,9.62) |  | 120.27(88.43,145.21) | 1.86(1.36,2.24) |  | -81.98(-87.47,-76.67) | -5.47(-5.80,-5.13) |
| Sri Lanka | 789.82(502.73,1099.98) | 14.27(9.09,19.88) |  | 317.52(226.62,455.99) | 6.22(4.44,8.93) |  | -59.80(-74.19,-17.60) | -2.23(-2.70,-1.76) |
| Sudan | 11524.04(3313.80,19247.22) | 129.59(37.27,216.44) |  | 5609.32(3674.69,7832.10) | 33.81(22.15,47.21) |  | -51.33(-69.00,26.63) | -3.95(-4.23,-3.67) |
| Suriname | 23.21(16.97,29.89) | 17.81(13.03,22.95) |  | 13.76(8.97,19.79) | 9.60(6.26,13.81) |  | -40.73(-61.27,-10.39) | -1.86(-1.99,-1.73) |
| Sweden | 132.72(112.19,148.80) | 8.59(7.26,9.63) |  | 20.83(14.19,32.85) | 1.14(0.78,1.80) |  | -84.31(-89.19,-71.97) | -5.17(-5.79,-4.54) |
| Switzerland | 153.10(124.05,171.07) | 13.25(10.73,14.80) |  | 33.99(27.04,41.05) | 2.55(2.03,3.08) |  | -77.80(-83.05,-70.52) | -4.79(-5.37,-4.20) |
| Syrian Arab Republic | 3003.55(1699.81,4010.04) | 50.72(28.70,67.71) |  | 339.69(250.70,457.03) | 9.27(6.84,12.48) |  | -88.69(-92.65,-75.27) | -4.61(-5.15,-4.07) |
| Taiwan (Province of China) | 479.00(443.42,510.30) | 8.70(8.05,9.26) |  | 84.43(68.24,100.18) | 2.87(2.32,3.40) |  | -82.37(-85.79,-78.31) | -3.87(-4.33,-3.42) |
| Tajikistan | 303.26(230.59,446.85) | 13.06(9.93,19.24) |  | 371.02(215.28,778.05) | 10.35(6.01,21.71) |  | 22.35(-36.44,175.17) | 0.34(-0.07,0.75) |
| Thailand | 2498.19(1729.19,3595.65) | 14.82(10.26,21.33) |  | 502.36(343.40,641.63) | 5.14(3.52,6.57) |  | -79.89(-86.27,-68.47) | -3.44(-3.64,-3.24) |
| Timor-Leste | 208.68(56.37,332.76) | 62.74(16.95,100.04) |  | 122.92(82.35,174.13) | 23.61(15.82,33.45) |  | -41.10(-61.66,74.12) | -3.58(-3.83,-3.33) |
| Togo | 478.99(126.45,724.65) | 27.18(7.17,41.11) |  | 407.94(261.93,580.88) | 12.33(7.92,17.55) |  | -14.83(-43.48,121.24) | -2.14(-2.34,-1.94) |
| Tokelau | 0.08(0.05,0.11) | 13.71(7.96,18.36) |  | 0.12(0.09,0.18) | 31.76(22.73,45.46) |  | 50.54(-7.39,243.11) | -1.66(-3.39,0.10) |
| Tonga | 3.98(2.51,5.59) | 9.53(6.01,13.36) |  | 2.07(1.40,3.21) | 5.31(3.59,8.24) |  | -48.00(-67.63,-1.30) | -1.66(-1.92,-1.41) |
| Trinidad and Tobago | 61.63(51.79,73.19) | 15.17(12.75,18.01) |  | 30.08(22.42,39.67) | 11.04(8.23,14.56) |  | -51.19(-65.59,-28.66) | -0.21(-0.71,0.29) |
| Tunisia | 1620.24(750.52,2280.35) | 52.17(24.17,73.43) |  | 246.41(182.69,328.68) | 8.91(6.61,11.88) |  | -84.79(-90.85,-65.39) | -4.74(-5.05,-4.43) |
| Turkey | 14833.77(6547.19,22850.71) | 72.40(31.96,111.53) |  | 1710.46(1286.98,2200.23) | 9.24(6.95,11.88) |  | -88.47(-93.60,-71.43) | -6.12(-6.35,-5.90) |
| Turkmenistan | 266.47(203.22,339.91) | 17.75(13.54,22.65) |  | 313.33(221.56,413.73) | 20.56(14.54,27.15) |  | 17.59(-11.19,57.18) | 2.59(1.76,3.44) |
| Tuvalu | 1.61(0.45,2.52) | 46.26(12.81,72.57) |  | 0.38(0.25,0.55) | 10.21(6.80,14.65) |  | -76.31(-86.21,-28.80) | -4.56(-4.83,-4.29) |
| Uganda | 2302.68(619.45,4491.57) | 27.35(7.36,53.35) |  | 2465.49(1485.78,4080.60) | 12.43(7.49,20.57) |  | 7.07(-28.05,186.33) | -2.23(-2.45,-2.02) |
| Ukraine | 1896.61(1621.42,2243.82) | 16.67(14.25,19.73) |  | 326.27(262.36,401.84) | 5.14(4.13,6.33) |  | -82.80(-86.90,-77.75) | -2.82(-3.42,-2.22) |
| United Arab Emirates | 168.88(111.97,231.42) | 28.65(19.00,39.26) |  | 54.96(31.54,78.69) | 4.11(2.36,5.88) |  | -67.45(-79.55,-36.54) | -4.07(-4.71,-3.43) |
| United Kingdom | 875.42(826.43,949.05) | 8.02(7.57,8.69) |  | 261.27(213.71,328.57) | 2.22(1.81,2.79) |  | -70.16(-75.83,-62.75) | -3.32(-3.72,-2.92) |
| United Republic of Tanzania | 4298.85(1068.91,8189.33) | 35.60(8.85,67.82) |  | 3611.22(2008.45,6365.23) | 14.80(8.23,26.08) |  | -16.00(-48.04,135.73) | -2.33(-2.56,-2.10) |
| United States of America | 4445.53(3831.71,4874.48) | 7.95(6.85,8.72) |  | 1473.01(1220.43,1847.90) | 2.48(2.05,3.11) |  | -66.87(-73.37,-55.10) | -3.21(-3.43,-2.98) |
| United States Virgin Islands | 3.98(3.03,4.92) | 12.45(9.48,15.41) |  | 0.37(0.22,0.64) | 2.75(1.61,4.80) |  | -90.75(-94.81,-82.12) | -4.49(-4.72,-4.26) |
| Uruguay | 119.72(97.35,141.74) | 14.62(11.89,17.32) |  | 40.65(31.22,51.32) | 6.16(4.73,7.78) |  | -66.04(-75.91,-55.86) | -2.61(-2.91,-2.31) |
| Uzbekistan | 1123.21(925.44,1317.80) | 13.13(10.82,15.40) |  | 2203.87(1577.07,2889.53) | 21.84(15.63,28.63) |  | 96.21(41.83,162.73) | 2.77(1.94,3.60) |
| Vanuatu | 13.24(5.41,18.83) | 19.44(7.94,27.65) |  | 12.62(8.00,17.35) | 10.83(6.86,14.89) |  | -4.63(-33.13,68.18) | -1.84(-2.25,-1.42) |
| Venezuela (Bolivarian Republic of) | 1006.36(901.98,1110.67) | 14.19(12.71,15.66) |  | 890.23(624.43,1201.28) | 13.44(9.43,18.14) |  | -11.54(-38.04,20.66) | 0.59(0.38,0.81) |
| Viet Nam | 3164.95(1315.14,4373.49) | 11.94(4.96,16.50) |  | 1107.78(719.19,1640.73) | 4.47(2.90,6.63) |  | -65.00(-79.17,-10.79) | -2.23(-2.63,-1.83) |
| Yemen | 8021.48(2297.43,12969.86) | 113.07(32.38,182.82) |  | 5136.73(3311.73,6997.78) | 37.25(24.02,50.75) |  | -35.96(-57.94,57.48) | -3.42(-3.69,-3.14) |
| Zambia | 1240.20(333.48,2383.65) | 33.03(8.88,63.49) |  | 942.57(568.23,1656.85) | 11.40(6.87,20.03) |  | -24.00(-61.85,214.96) | -3.02(-3.34,-2.70) |
| Zimbabwe | 369.04(266.43,484.37) | 7.66(5.53,10.06) |  | 494.16(326.76,699.37) | 7.85(5.19,11.11) |  | 33.91(-1.96,94.58) | 0.71(0.42,1.01) |
